# Assessment of Immunological Potential of Glial Restricted Progenitor Graft In Vivo—Is Immunosuppression Mandatory?

**DOI:** 10.3390/cells10071804

**Published:** 2021-07-16

**Authors:** Urszula Kozlowska, Aleksandra Klimczak, Karolina Anna Bednarowicz, Tomasz Zalewski, Natalia Rozwadowska, Katarzyna Chojnacka, Stefan Jurga, Eytan R. Barnea, Maciej K. Kurpisz

**Affiliations:** 1 Hirszfeld Institute of Immunology and Experimental Therapy, Polish Academy of Sciences, 53-114 Wroclaw, Poland; k.szurula@gmail.com (U.K.); aleksandra.klimczak@hirszfeld.pl (A.K.); 2 Institute of Human Genetics, Polish Academy of Sciences, 60-479 Poznan, Poland; karolina.bednarowicz@igcz.poznan.pl (K.A.B.); natalia.rozwadowska@igcz.poznan.pl (N.R.); 3 NanoBioMedical Centre, Adam Mickiewicz University, 61-614 Poznan, Poland; tomekz@amu.edu.pl (T.Z.); stjurga@amu.edu.pl (S.J.); 4 Institute of Bioorganic Chemistry, Polish Academy of Sciences, 61-704 Poznan, Poland; kchojnacka@ibch.poznan.pl; 5 The Society for the Investigation of Early Pregnancy (SIEP), Cherry Hill, NJ 08003, USA; barnea@earlypregnancy.org; 6 BioIncept LLC, Cherry Hill, NJ 08003, USA

**Keywords:** amyotrophic lateral sclerosis, ALS, SOD1G93A, histocompatibility, transplantation, immunosuppression, CNS, neurodegenerative disorder

## Abstract

Amyotrophic lateral sclerosis (ALS) is an incurable neurodegenerative disease, causing motor neuron and skeletal muscle loss and death. One of the promising therapeutic approaches is stem cell graft application into the brain; however, an immune reaction against it creates serious limitations. This study aimed to research the efficiency of glial restricted progenitors (GRPs) grafted into murine CNS (central nervous system) in healthy models and the SOD1G93A ALS disease model. The cellular grafts were administered in semiallogenic and allogeneic settings. To investigate the models of immune reaction against grafted GRPs, we applied three immunosuppressive/immunomodulatory regimens: preimplantation factor (PiF); Tacrolimus; and CTLA-4, MR1 co-stimulatory blockade. We tracked the cells with bioluminescence imaging (BLI) in vivo to study their survival. The immune response character was evaluated with brain tissue assays and multiplex ELISA in serum and cerebrospinal fluid (CSF). The application of immunosuppressive drugs is disputable when considering cellular transplants into the immune-privileged site/brain. However, our data revealed that semiallogenic GRP graft might survive inside murine CNS without the necessity to apply any immunomodulation or immunosuppression, whereas, in the situation of allogeneic mouse setting, the combination of CTLA-4, MR1 blockade can be considered as the best immunosuppressive option.

## 1. Introduction

Amyotrophic lateral sclerosis (ALS) is a civilization neurodegenerative disease that affects upper and lower motor neurons (UMN and LMN, respectively) in various proportions. It is also known as Lou Gehrig disease or less commonly as motor neuron disease. It affects 1–3 in 100,000 people. The mean age at diagnosis is 56 years old. The likelihood of the disease occurrence increases with age [1]; however, there are also reports of ALS occurring in younger people in their 20 s [2]. There are two onsets of ALS-limb onset (weakness, stumbling, weak handgrip) and the bulbar onset (dysphagia, dysarthria); the second is more typical for females. Progressive dysfunction of the motor neurons leads to skeletal muscle weakness and atrophy. The first symptoms (first phase) are muscle weakness, cramps, and fasciculations, which progress into an inability to walk, take manual actions, speak, and eat (second phase). In the last phase of the disease, there is a loss of intercostal muscles, leading to respiratory failure and death-most commonly within 2–3 years after diagnosis [3]. Although ALS is considered a motor neuron disease, there are cases of frontotemporal dementia (FTD) most possibly associated with the abnormalities within the C9orf72 (chromosome 9 open reading frame 72) gene, which contains repeat expansion of hexanucleotide GGGGCC [4,5]. About 10% of ALS cases are reported to be familial ALS (FALS)-individuals with ALS history in their families-while the others (about 90% of cases) are sporadic (SALS). Many genes are mutated or expressed abnormally, such as SOD1, TARDBP, C9orf72, FUS/TLS, and many others recently reported [1,3]. Still, there are no clearly pinpointed environmental risk factors for sporadic ALS; however, it is speculated that it might involve psychological stress, too harsh physical training routine, branched-chain amino acids (BCAAs) supplementation, environmental pollution, cigarettes smoking, and others [6,7].

To date, the perspectives for effective treatment are relatively poor-there is no treatment protocol approved that might successfully cure ALS. Riluzole’s only registered pharmacologic drug might extend ALS affected individuals’ lives for approximately 2–3 months [8]. The application of stem cells is a prospective therapy currently being tested. Because of the complexity of the brain structure and the neuron itself, the application of cell transplants is limited. However, there were promising results reported after human bone marrow-derived mesenchymal stem cells (MSCs) were administered intrathecally into a mouse model of ALS [9]. It had been previously described that the site of transplantation of neural progenitor cells plays a critical role in graft survival [10]. Therefore, it was suggested that, when considering the stem cell therapy for ALS patients, the graft should be administrated intrathecally-as less invasive for the patient than the intrastriatal administration, but still giving cellular grafts a higher chance to survive behind the blood and brain barrier (BBB). There is, however, a major concern, when considering the therapeutic application of stem cells in neurodegenerative diseases-based on the question: “What is the ratio between their real therapeutic potential to possible risk of complications?”-and those complications might be the following: the risk of tumorigenesis, infections, and the possibility to start unwanted acute inflammatory response within central nervous system (CNS).

The CNS is a system characterized by very specific immunology; therefore, predicting its reaction to cellular graft is very difficult [11,12]. Since the discovery of CNS-draining lymphatic vessels, called the glymphatic system, more highlights were introduced on the blood–brain barrier gap [13,14]. It is suggested that pathogen-associated molecular patterns (PAMP) and danger-associated molecular patterns (DAMPs) collected in the brain travels through this system into peripheral lymph nodes, where they are presented to naïve T-cells, and then, when necessary, infiltrating activated T-cells might cross blood and brain barrier. When this happens, it is suggested that the microglial cells sometimes might actually play a more anti-inflammatory role inside the brain, regulating its homeostasis by reaching microglia–T-cell equilibrium and, in consequence, supporting the maintenance of pro-regenerative character of the local environment [13].

In this study, we tried to assess the potential immunogenicity of murine glial restricted progenitors (GRPs) transplanted directly into cisterna magna of ALS B6SJL-Tg(SOD1*G93A)1Gur/J male mouse model and involvement of neuroimmune response to GRPs administration in semiallogeneic (DBA1/J), allogeneic (C57BL/6), and immunodeficient (SCID) male mouse model. Neuroimmune response to GRPs was studied under immunosuppressive (Tacrolimus or CTLA-4 MR1 co-stimulatory blockade) and immunomodulatory (preimplantation factor) protocols [15,16].

## 2. Materials and Methods

### 2.1. Cell In Vitro Culture

Murine GRP cells were received as a gift from the Department of Radiology and Radiological Science, Johns Hopkins School of Medicine (Baltimore, MD, USA). Cells were isolated as previously described by Srivastava, Bulte et al. [17] from spinal cords dissected from ^Luc+/PLP/GFP+^ mice between E12.5 and E14 stage. Tissue samples were plated on a Petri dish in DMEM/F12 medium (Thermo Fisher, Waltham, MA, USA), then incubated in pre-warmed TrypLE Express (Thermo Fisher, Waltham, MA, USA) with 10 mg/mL DNase-1 (A&A Biotechnology, Gdansk, Poland) for 10–12 min and incubated for a further 10 min. Next, 5 mL of GRP medium was added and centrifuged at 1000 rpm for 5 min. The cell pellet was resuspended in 10 mL of GRP medium with 10 mg/mL of DNase, and incubated at 37 °C in a humidified incubator with 5% CO_2_ for 10 min. The pellet was then mechanically agitated and centrifuged at 1000 rpm for 5 min; resuspended in 10 mL of GRP medium (DMEM/F12 medium (Thermo Fisher, Waltham, MA, USA); supplemented with N2 and B27 (Life Technologies, Carlsbad, CA, USA), 1% BSA (Abcam Cambridge, Great Britain), Penicillin-streptomycin (Life Technologies, Carlsbad, CA, USA), and 20 ng per mL bFGF (Peprotech, Rocky Hill, NJ, USA)); and plated on coated with poly-L-lysine and laminin (Sigma, Saint Louis, MI, USA) 25 mL flasks in a humidified incubator at 37 °C with 5% CO_2_.

### 2.2. GRPs Characteristics

The cells were cultured until a monolayer and 80% of confluency was achieved, then detached from culture flasks and characterized by immunofluorescence staining and flow cytometry. A detailed description of the staining procedures is provided in previous paper [18]. All the antibodies and isotype controls used are neural markers specific for GRP cells (see Table 1). For each antibody, depending on its isotype and the fluorochrome conjugated, the proper isotype control staining was prepared for all types of analyzed cells. The dilutions of each primary antibody and isotype controls used are presented in Table 1. Samples were analyzed with an Amnis cytometer. Data were analyzed using IDEAS Application software v 6.0. Immunostainings were analyzed using a fluorescent microscope (Leica, Wetzlar, Germany) equipped with a monocamera and LAS X software for picture acquisition.

### 2.3. GRP Genotyping

Assessment of the cell immunological haplotype was done according to the protocol described below [19]. Genomic DNA was isolated from the mouse tail tips (BALB/cJ (RRID: IMSR_JAX:000651); C57BL/6J, DBA/1J) according to Hofer et al. DNA was isolated from msGPR cells with Quiamp DNA blood mini kit (Qiagen, Hilden, Germany) according to manufacturer’s manual. Then, 40 ng of genomic DNA was amplified in 25 µL PCR reaction mixture (PCR Master Mix, Promega, Madison, WI, USA) with forward (kb25 5′-GGCTCTCACACTATTCAGGT-3′; kk5 5′-GGCTCTCACACGTTCCAACG-3′) or reverse (kb33 5′-GCGTCGCGTTCCCGTTCTT-3′; kk23 5′CTCCAGGTAGGCCCGGTC-3′) primers. For H2K-multiplex, all four primers were used. Gel electrophoresis was performed on 2.0% agarose gel and visualized with ethidium bromide under a UV lamp (Uvitec, Cambridge, UK).

### 2.4. Cell Graft Preparation

GRP cells were detached from the culture flask with TrypLE (Thermo Fisher, Waltham, MA, USA) enzymatic digestion. The reaction was disrupted by adding PBS in 1:3 proportion. Cells were washed two times with PBS and centrifuged at 1000 rpm for 5 min. The viability of the GRPs was assessed with trypan blue exclusion, and only suspensions with over 70% of cell viability were used for transplantation. The suspension was prepared of 0.5 mln GRP cells in 10 µL of PBS.

### 2.5. Experimental Groups

**Figure 1:**
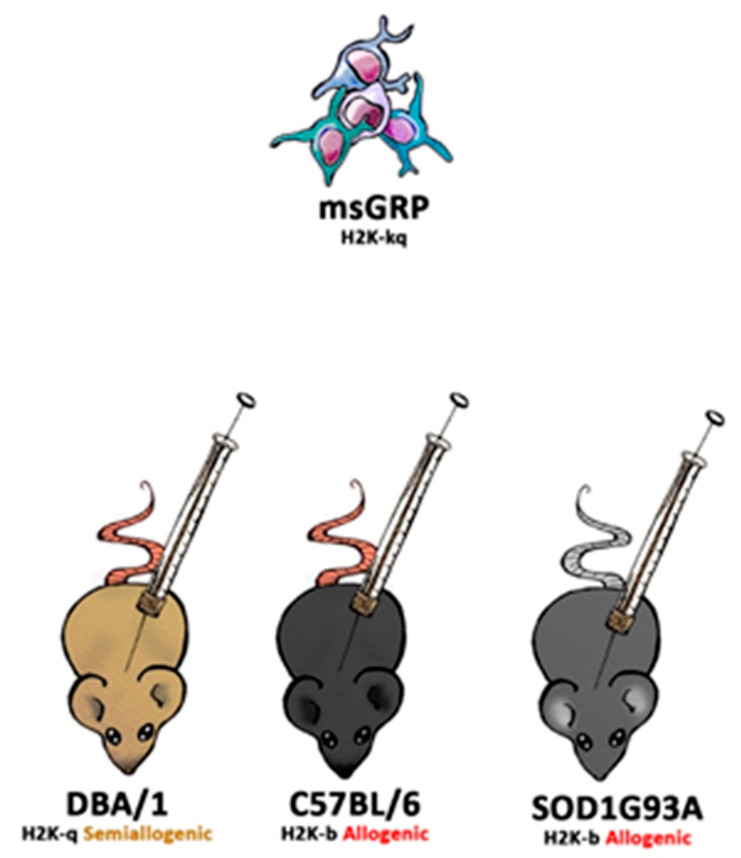
The scheme of in vivo murine glial restricted progenitors (GRPs) grafting variants.

DBA1/J (RRID: IMSR_JAX:000670), C57BL/6J (RRID: IMSR_JAX:000664), B6SJL-Tg (SOD1*G93A)1Gur/J (RRID: IMSR_JAX:004435) and SCID (RRID: IMSR_JAX:001303) mice were obtained from Jackson Laboratory (Bar Harbor, ME, USA). Animals were hosted (with approval of Local Bioethical Committee; 12/2017) in humanitarian conditions. Animals of each strain (except SCID-control where no immunosuppression applied variant was used) were divided into five groups. Group 1: sham surgery (C57BL/6 *n* = 6, DBA1/J *n* = 6, SOD1G93A *n* = 6), group 2: no immunosuppression applied (C57BL/6 *n* = 4, DBA1/J *n* = 5, SOD1G93A *n* = 6, SCID *n* = 5), group 3: preimplantation factor was applied (C57BL/6 *n* = 5, DBA1/J *n* = 5, SOD1G93A *n* = 6), group 4: Tacrolimus (FK506) was applied (C57BL/6 *n* = 4, DBA1/J *n* = 4, SOD1G93A *n* = 6), and group 5: costimulatory blockade MR1 + CTLA4 was applied (C57BL/6 *n* = 4, DBA1/J *n* = 4, SOD1G93A *n* = 6) (Figure 1).

### 2.6. Immunosuppressive and Immunomodulatory Drug Administration

Preimplantation factor (Bio-Synthesis, Inc., Lewisville, TX, USA) was applied in the amount of 1 mg/kg body weight starting at day −1 prior to graft administration and administered for 2 weeks after GRPs’ transplantation according to Azar and Shainer et al. [20]. Tacrolimus (Sigma Aldrich, St. Louis, MI, USA) was applied in the amount of 1 mg/kg body weight starting at day −1 prior to graft administration and administered every day until the end of observation. Co-stimulatory blockade MR1 (InVivoMab anti-mouse CD154 (CD40L, Hozel Diagnostika, Bio X Cell, USA; RRID: AB_1107601)) + CTLA4 (ORENCIA^®^(Abatacept), Bristol-Myers Squibb Company, New York, NY, USA; CAS no. 332348-12-6) was administered at an amount of 25 mg/kg on days 0, 2, 4, and 6.

### 2.7. Cell Transplantation

Before the operation, mice were anesthetized with 5% (induction) and maintained in 2% isoflurane. Animals were immobilized in a stereotaxic device (Leica, Wetzlar, Germany). Then, 10 µL of cell suspension was administered into cisterna magna 2 µL/min via an infusion pump (KD Scientific, Hollston, MA, USA). Details of the procedure were previously described by Habish and Janowski et al. [21].

### 2.8. Serum Collection

Every week, starting from the day of graft administration (day 0), ~100 µL of blood was collected from the mouse tail. Blood was stored 1 h at room temperature, then overnight at +4 °C. Serum was gently aspirated and centrifuged 10 min at 2000 rpm to separate blood morphotic bodies. Purified serum was stored in −80 °C until further analysis.

### 2.9. Cerebrospinal Fluid (CSF) Collection

CSF was collected on days 0, 7, 10, 14, and 28 for the C57BL/6 model and on days 0, 7, 10, 28, and 63 for the DBA1 model. In the SOD1G93A model, CSF was aspirated on day 0 and at the end of the procedure. Moreover, 3–7 µL of fluid volume was collected with the handmade device from cisterna magna and stored at −80 °C. Only CSF water-like transparent fluid was qualified for further ELISA tests.

### 2.10. Magnetic Resonance Imaging (MRI) In Vivo

All animal experiments were performed according to respective local regulations and were approved by the Local Ethical Committee for Animal Experimentation, Poznan University of Life Sciences (Permission No 12/2017). The experiments were performed on SCID, C57BL/6, and DBA1 mouse strains before implementing SPIO labeled cells into cisterna magna as a reference and then after administration of SPIO labeled cells into cisterna magna. Mice were MRI scanned 1 day after cell transplantation. The mouse was anesthetized with 2% isoflurane (Baxter, Deerfield, IL, USA) in a 50% oxygen and 50% air mixture. An experimental animal was placed at the MRI-compatible mouse dedicated bed. The head MR images were performed on a 9.4 T Agilent MRI scanner (400 MHz resonance frequency for protons) using a volume Millipede Coil-40 mm diameters (Agilent, Santa Clara, CA, USA). The imaging protocol consisted of spin’s density and T2-weighted MRI using Fast Spin Echo technique (FSEMS: FOV 19 × 19 mm, Matrix 256 × 256, TR = 8000 ms, TEff for spin’s density = 10 ms, TEff for T2-weighted = 40 ms, slices = 10, 75 × 75 in-plane resolution, slice thickness = 1 mm, NA = 2, scan time ~8 min).

### 2.11. Bioluminescent Imaging (BLI) of Graft Survival

Imaging was performed on days 0, 3, and 7, and then every week. For accuracy, the imaging was also performed on the 10th day after grafting. Measurements were made with IVIS Lumina LT series III (Perkin Elmer, Waltham, MA, USA) and Xeno Light Luciferin (Perkin Elmer, Waltham, MA, USA) at 150 mg per kg. Time optimal enzyme activity was set experimentally: 10 min for SCID, 10 min for C57BL/6, 7 min for DBA1, and 10 min for SOD1G93A mouse strains.

### 2.12. Multiplex ELISA Assay

Multiplex ELISA 23-cytokine kit (Cat# M60009RDPD, RRID: AB_2857368) was obtained from Bio-Rad (Hercules, CA, USA). The multiplex ELISA test was done according to Bio-Rad protocol. Samples were thawed on ice and diluted in sample diluent provided in the kit (1:4 for serum and in 1:10–1:20 range for CSF, depending on the volume of the type of fluid that was collected). Then, 50 µL samples were added into wells of a 96-well plate as a template. The template 96-well plate was stored on ice. Measurement standards were used, diluted nine times according to the protocol, and added to the template plate. Properly mixed beads were added to the 96-well plate assay. The plate was washed three times with washing buffer, 50 µL samples, standards, and blank were transferred to the measurement plate by multichannel pipette. The plate was sealed with tape and incubated for 30 min. Afterwards, the plate was rewashed three times. Then, 25 µL of detection antibody was added to the microwells with a multichannel pipette. The plate was sealed and incubated for 30 min, and then washed three times using washing buffer. Then, 50 µL per well of Streptavidin-PE was added with a multichannel pipette. The plate was sealed and incubated for 10 min. Wells were washed three times with washing buffer. Beads were resuspended with 125 µL of assay buffer, and analysis was performed with Bio-Plex 200 reader.

### 2.13. Tissue Collection

When the BLI signal from the cell graft was not detected at two consecutive time points, mice under general anesthesia were subjected to whole-body perfusion, with 20% sucrose and 10% buffered formalin suitable for histology assays (Sigma, Saint Louis, MI, USA). Then, the head with the spine was separated and incubated in 10% buffered formalin for 24 h. After this time, the brain and spinal cord were isolated, paraffin fixed, and cut into histological slides.

### 2.14. Immunohistochemical Stainings

Slides were deparaffinized with xylene and a descending gradient of alcohol of 100%, 70%, and 30% respectively, for 10 min at each solution. Epitopes were exposed via 20 min incubation in 97 °C of citric buffer, pH = 6, supplemented with 0.01% Tween 20. Next, tissue slides were incubated in 10% goat serum and 1% BSA to minimize the risk of an unspecific reaction. The distribution of antibodies used in immunostaining protocol was as follows: first primary antibody, anti-GFP (Abcam, Cambridge, UK, Cat# ab183734, RRID:AB_2732027; 1:500, +4 °C, overnight); then green fluorescent secondary antibody (Alexa Fluor 488 nm, Abcam, Cambridge, UK, Cat# ab150077, RRID:AB_2630356; 1:700, 1 h, room temperature); then another set of primary antibody included the following: anti-CD3 (Abcam, Cambridge, UK, Cat# ab16669, RRID:AB_443425; 1:150, 1 h, room temperature), anti-CD45 (Abcam, Cambridge, UK, Cat# ab10558, RRID:AB_442810; 1:200, 1 h, room temperature), anti-TMEM119 (Abcam, Cambridge, UK, Cat# ab209064, RRID:AB_2800343; 1:1000, +4 °C, overnight), anti-MCP-1 (Thermo Fisher, Waltham, MA, USA, Cat# MA5-17040, RRID:AB_2538512; 1:200, 1 h, room temperature), or anti-TLR-4 (Abcam, Cambridge, UK, Cat# ab13556, RRID:AB_300457; 1:200, 1 h room temperature); and then red fluorescent secondary antibody (Alexa Fluor 594 nm, Abcam, Cambridge, UK, Cat# ab150080, RRID:AB_2650602; 1:700, 1 h, room temperature) was applied. In between those steps, triple washing in PBS was performed. Cell nuclei were stained with DAPI (Vector Laboratories, Burlingame, CA, USA, Cat# H-1200, RRID: AB_2336790).

### 2.15. Data Analysis and Statistics

From the data obtained in BLI observations, a background measurement was subtracted. The first result (measurement at day 1) was normalized to 100%. Then, a proportion of signal intensity obtained in the following measurements was calculated, using a measurement at day 1 (100%) as a reference. The graft was classified as rejected when less than 3% of the original signal was measured at two consecutive measurements.

*p*-values were calculated using *t*-test assuming unequal variances with Welch correction for graft viability comparing each group (variant) with each other. The *p*-value for multiplex ELISA was calculated using one-way analysis of variance (ANOVA).

## 3. Results

### 3.1. Mouse GRPs’ Expression of TLR-4

**Figure 2:**
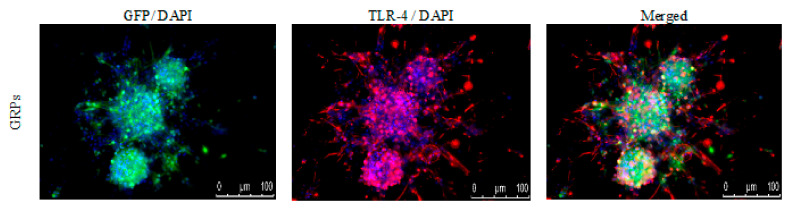
Mouse GRPs’ expression of toll-like receptor-4 (TLR-4): The immunofluorescent (IF) staining of TLR-4 revealed that murine GRP is characterized by strong expression of TLR-4 receptor, which may lead to intensification of co-stimulatory molecules expression. Abbreviations: GRP-glial-restricted progenitor cell.

The immunological characteristics of the mouse GRPs were thoroughly described in our previous study [18]; however, to better understand prospective cellular interactions, expression of TLR-4 (toll-like receptor-4) was repeatedly performed. It appeared that murine GRPs display TLR4 markers on their cell surface (Figure 2), which in certain situations, when stimulated, may lead to activation of immune response to PAMPs and/or DAMPs and intensification of expression of co-stimulatory molecules.

### 3.2. MRI Evaluation of the GRPs’ Posttransplant Localization in CNS

**Figure 3:**
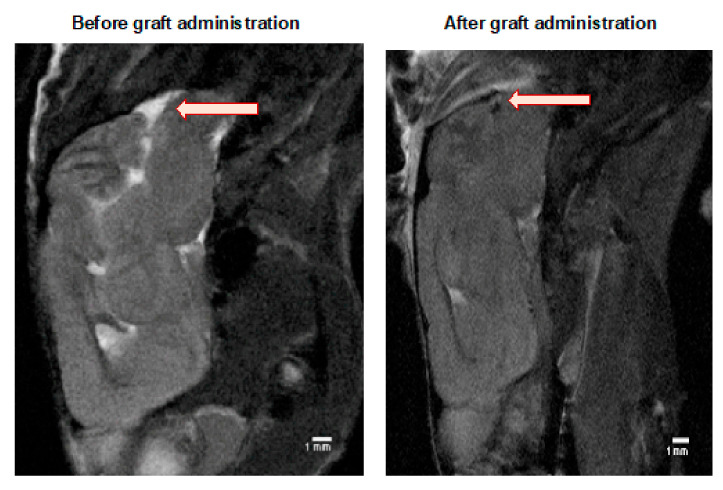
MRI evaluation of the GRPs’ post-transplant localization in CNS: The MRI scan image of the representative (SCID) mouse at the day before (left picture) and a day after (right picture) of murine GRP transplantation into cisterna magna. The black dot (arrow), visible on the right picture shows the localization of SPIO-labelled cells. Abbreviations: GRP-glial-restricted progenitor cell; MRI-magnetic resonance imaging; SPIO-superparamagnetic iron oxide.

The MRI scanning was performed in selected mice to ensure that the cell graft was inserted correctly into a cisterna magna space (Figure 3). Murine GRPs were previously labeled (for 24 h) with SPIO (small paramagnetic iron oxide). Then, on day 1+ after transplantation, a signal was revealed by MRI scan indicating proper localization of the cells, not touching the brain tissues as its damage could elicit an immune response.

### 3.3. Murine GRP Haplotype Evaluation

**Figure 4:**
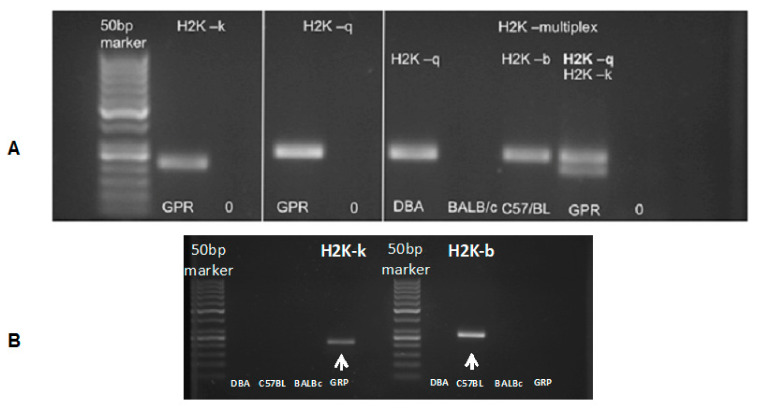
Murine GRP haplotype evaluation: (**A**) (in left panels) presents H2K-k and H2K-q typing results of the murine GRPs. (**A**) (in the right) presents the evaluation of the haplotype of DBA/1 mice (H2K-q), BALB/c (H2K-d), and C57BL/6 (H2K-b), as well as tested murine GRP cells, which gave positive results for both H2K-q and H2K-k haplotype. (**B**) (below) presents PCR results using only H2K-b specific primers to rule out the possibility of H2K-b expression, which is characterized with similar to H2K-k weight in the gel. Abbreviations: GRP-glial-restricted progenitor cell; PCR-polymerase chain reaction.

The murine GRP cells obtained revealed an H2K-kq haplotype (Figure 4). The PCR result additionally confirmed the data generated by DNA sequencing (data not shown). The results indicated that the murine GRPs obtained had allogenic character regarding the graft in the C57BL/6 model and semiallogenic character when transplanted to DBA/1 mouse.

### 3.4. Immunological Properties of GRP-In Vivo Observations in the Semiallogenic Model

**Figure 5:**
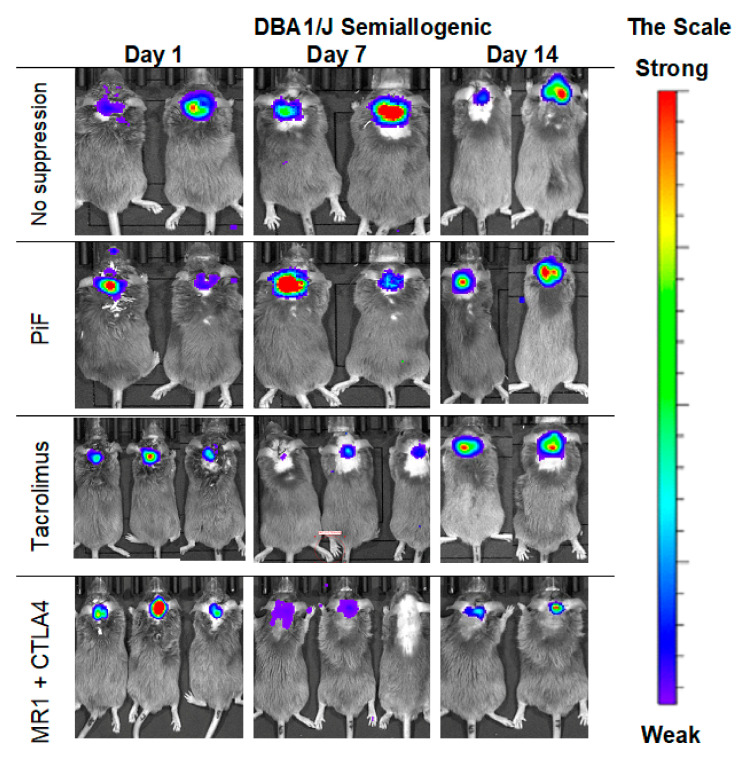
Representative pictures of the murine GRP graft survival after transplantation into semiallogenic DBA1 mouse model: The BLI signal from the graft was measured on days 7 and 14 post-transplantation. Abbreviations: BLI-bioluminescence imaging; GRP-glial-restricted progenitor cell.

The observations of the cell graft survival when assessed with the BLI technique revealed that the graft survives the longest time when applied in a semiallogenic model. The images (Figure 5) present the BLI measurement in the first two weeks of the experiment. The results show that the signal increases on day 7 post-transplantation when no immunosuppression or only PiF was applied. However, the signal was weaker on day 7 when Tacrolimus or co-stimulatory blockade were used. However, the bioluminescent signal was detected again after 2 weeks post-transplantation, suggesting that the graft survived in all applied immunomodulatory regimens.

### 3.5. Immunological Properties of GRPs-In Vivo Observation in the Allogenic Model

**Figure 6:**
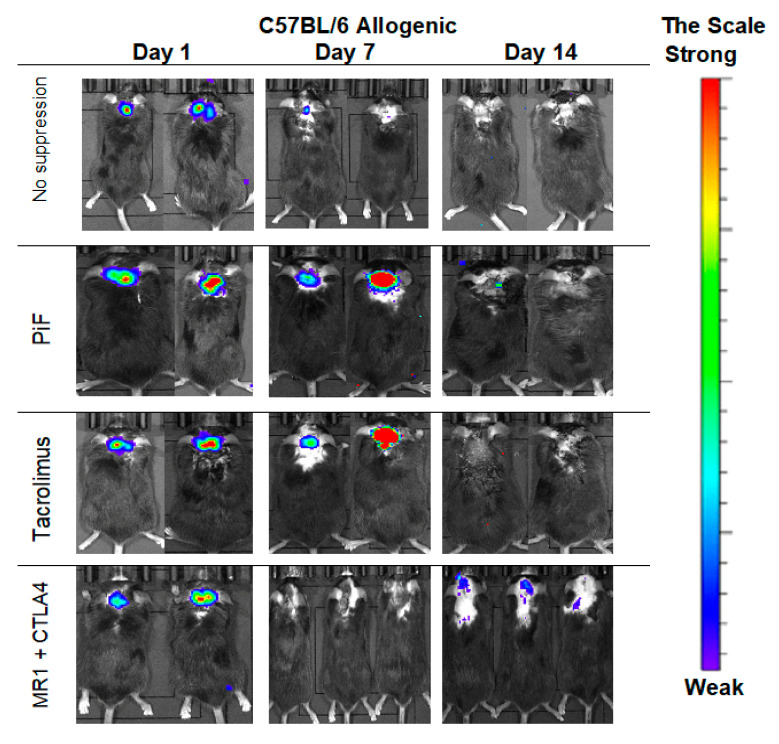
Representative pictures illustrating the murine GRP graft survival after transplantation into an allogeneic C57BL/6 mouse model: The BLI signal vanished quickly at the day 7 in immunosuppression-free and was weak at the co-stimulatory blockade group; surprisingly, however, the strongest signal intensity on day 14 was revealed again in the co-stimulatory blockade group. Abbreviations: BLI-bioluminescence imaging; GRP-glial-restricted progenitor cell.

The observations performed in the allogeneic C57BL/6 mouse model (Figure 6) showed that, after 7 days post-transplantation, the strongest signals were revealed in PiF- and Tacrolimus-applied groups of mice. The signals obtained on day 7 for the immunosuppression-free group and co-stimulatory blockade group were detectable. Surprisingly, after 14 days post-transplantation, the strongest signal occurred in a co-stimulatory blockade group.

### 3.6. Comparison of Graft Survival in Semiallogenic DBA1 and Allogenic C57BL/6 Mouse Models

**Figure 7:**
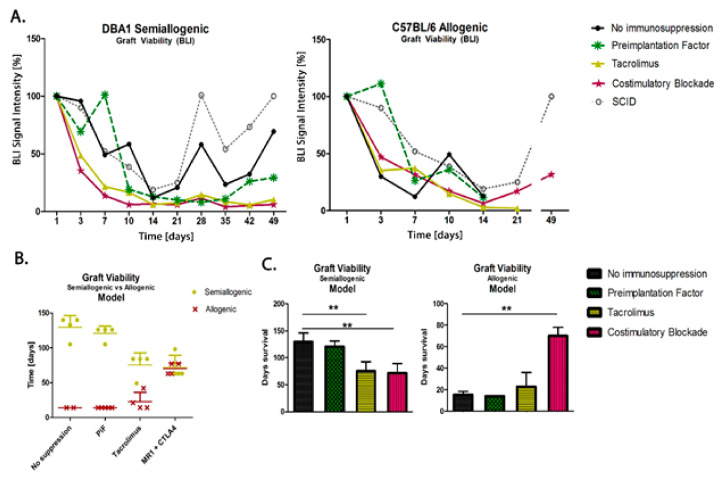
Comparison of graft survival in semiallogenic DBA1 and allogenic C57BL/6 mouse models: (**A**) Graphic presentation of GRP graft viability (presented as the proportion out of initial BLI as 100% of intensity signal) transplanted into healthy models in semiallogenic (left) and allogenic (right) combinations. (**B**) Graphic presentation of cellular graft viability in semiallogenic (yellow dots) and allogenic (red crosses) mouse models after performed GRP transplantation. The murine GRP graft survived well in semiallogenic mice even if no immunosuppression was applied. The graft survival in the allogenic model was the longest and comparable to the semiallogenic model after the co-stimulatory molecules blockade. (**C**) Bar graphics of graft survival in semiallogenic mouse model (right) as compared with different groups of immunosuppressive/immunomodulatory regimens (** *p* < 0.005). Unpaired Student’s *t*-test; data are presented as median, error bars: SD; the total number of biological replicates per group = 4–5. Abbreviations: BLI-bioluminescence imaging; GRP-glial-restricted progenitor cell.

The scatters presented in Figure 7 show the results of the measurements of murine GRP (H2K-kq) graft survival in semiallogenic (H2K-q) and allogenic (H2K-b) mouse models after 49 days (7 weeks) post-transplantation. The data show that the cells can survive this time without using immunosuppression when transplanted in semiallogenic conditions (the observation was not conducted over a longer period for the allogenic model because the collection of brain tissue was a priority at this time point for histological examination). In both models, the PiF regimen showed a good BLI signal in the first 10 days. The detected signal grew and exceeded 100% on day 7 in the semiallogenic and day 3 in the allogenic mouse model. Both Tacrolimus and co-stimulatory molecule blockade did not provide satisfactory results in the semiallogenic DBA1 mouse model. A similar effect was observed in the allogeneic C57BL/6 model, where, at day 7 post-transplantation, all applied immunomodulatory regimes provided better results than the immunosuppression-free variant; however, the signal in the Tacrolimus group decreased continuously and was subsequently lost on day 21. Finally, the signal intensity measured on day 49 post-transplantation in the allogenic model indicated the best detection of graft when the co-stimulatory molecules blockade was applied (Figure 7A). Interestingly, during observations of BLI signals, those were similarly dynamic in the semiallogenic model compared with the SCID mouse model (without own immunity). Both signals from SCID immunodeficient and DBA1 (semiallogenic) immunocompetent mouse were similar in the variant without immunosuppression applied at the indicated time period.

In the study on immunomodulatory and/or immunosuppressive regimes after applying the murine GRPs in semiallogenic and allogenic models, it has been clearly documented that the main histocompatibility complex (MHC) plays an essential role in the GRPs’ graft survival. After applying Tacrolimus, the overall survival of the cellular graft increased in the allogenic model, but decreased in the semiallogenic model; however, the results obtained in both models were similar after applying co-stimulatory blockade (Figure 7B). In the semiallogenic model, graft survived much longer without immunosuppression than in the allogenic model with co-stimulatory blockade (Figure 7C).

### 3.7. Cytokine Levels in Serum and CSF of DBA1 Semiallogenic Model

**Figure 8:**
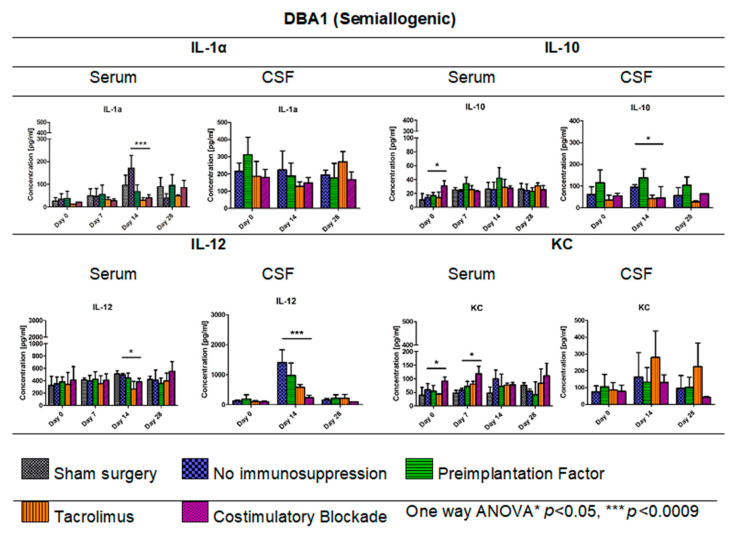
Cytokine levels in serum and CSF of DBA1 semiallogenic model: IL-1α, IL-10, IL12, and KC levels in serum and CSF samples were measured in semiallogenic DBA1 mice. There were significant differences between variants of the immunomodulatory regime in the level of IL-1α observed on day 14 (*p* < 0.0009). Significant differences were also found between the studied variants in serum samples for IL-10 (day 0, *p* < 0.05), IL-12 (day 14, *p* < 0.05), and KC levels (days: 0 and 7, *p* < 0.05). Significant differences in cytokine level in CSF were observed for IL-10 (*p* < 0.05) and IL-12 (*p* < 0.0009) on day 14 post-transplantation. Unpaired Student’s *t*-test; data are presented as mean, error bars: SD; the total number of samples: *n* = 77, total number per variant at each time point: *n* = 3–5. The total number of biological replicates per group: *n* = 5. Abbreviations: CSF-cerebrospinal fluid.

Figure 8 illustrates the levels of cytokines IL-1α, IL-10, IL-12, and KC (keratinocyte-derived chemokine) measured in serum and CSF of semiallogenic mouse model on days 0, 7, 14, and 28 for serum and days 0, 14, and 28 for CSF. The IL-1α level in serum of semiallogenic DBA1 model was the lowest in Tacrolimus and co-stimulatory blockade group; however, on day 28, IL-1α level in co-stimulatory blockade variant increased and was comparable to that detected in the PiF group. A significant difference (one-way ANOVA, *p* < 0.0009) as compared with the other groups was observed on day 14 posttransplantation, where IL-1α concentration was the highest in the serum of mouse groups without immunosuppression (171.25 pg/mL) versus the PiF (67.65 pg/mL) group. There was no significant difference between IL-1α concentration in CSF at the observation time; however, the lowest level was detected in Tacrolimus (128.29 pg/mL) and co-stimulatory blockade (148.04 pg/mL) on day 14, and was the highest in the PiF group on day 0 before the graft application (310.98 pg/mL). There was a significant difference in the serum IL-10 level at the starting point (day 0, one-way ANOVA, *p* < 0.05), and IL-10 was detected to be the highest in the co-stimulatory blockade group (30.92 pg/mL). Overall concentration in the other time points was relatively similar for all of the studied mice groups with this cytokine; however, the highest level of IL-10 was detected in the serum of PiF variant on day 14 post-transplantation (42.08 pg/mL). On day 14 in the PiF group, the highest level of IL-10 concentration detected in CSF (137.71 pg/mL) was also observed, one-way ANOVA *p* < 0.05. The lowest level of IL-10 in CSF was observed at each time point in groups when Tacrolimus and co-stimulatory blockade were applied. The level of IL-12 in serum was similar in each applied variant (groups), but slightly increased after transplantation. On day 14, the lowest level of IL-12 was found in the serum of Tacrolimus group (264.01 pg/mL,) one-way ANOVA *p* < 0.05. In CSF, IL-12 concentration was very low at the starting point (day 0); however, it increased on day 14 post-transplantation in the group where no immunosuppression was applied (1400.40 pg/mL). The lowest concentration of IL-12 in CSF at day 14 was observed in the co-stimulatory blockade group (235.17 pg/mL), one-way ANOVA overall *p* < 0.0009. On day 28, the IL-12 level in CSF of all groups returned to a similar level as detected on day 0. There were significant differences in serum KC level (one-way ANOVA *p* < 0.05) on day 0 between studied mice groups-the highest level was found in the co-stimulatory blockade group (91.26 pg/mL) and the lowest in the Tacrolimus group (43.01 pg/mL). At PiF and immunosuppression-free groups, the level of KC was similar. Another significant difference (one-way ANOVA *p* < 0.05) was observed on day 7, where the lowest concentration of KC in serum was assessed in the group without immunosuppression (57.70 pg/mL), and the highest in a co-stimulatory blockade group (118.43 pg/mL). There were no significant differences in KC levels in CSF, whereas its concentration was the highest on day 14 (279.35 pg/mL) and 28 (224.04 pg/mL) post-transplantation in the Tacrolimus group.

### 3.8. Cytokine Levels in Serum and CSF Samples in C57BL/6 Allogenic Mouse Model

**Figure 9:**
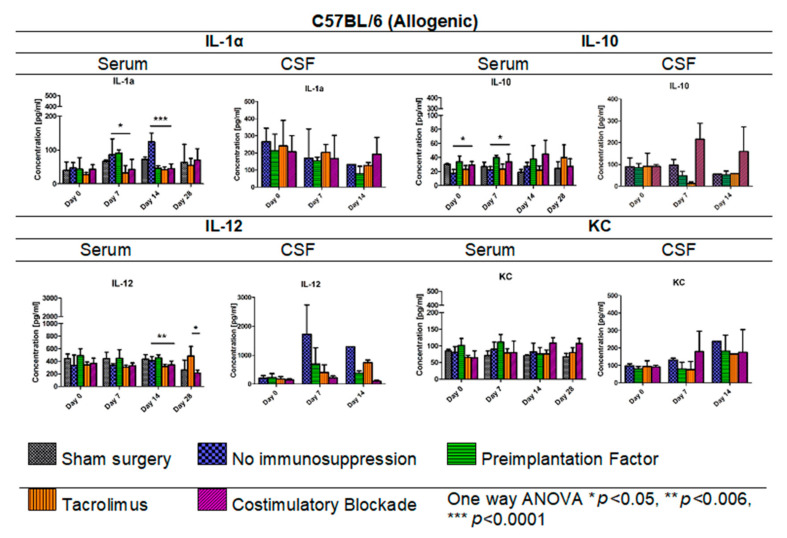
Cytokine levels in serum and CSF samples in C57BL/6 allogenic mouse model: IL-1α, IL-10, IL-12, and KC levels in serum and CSF samples were assessed in allogeneic mice. The most significant differences between variants under study (different immunomodulatory regime) were detected in serum with respect to IL-1α (*p* < 0.05 on day 7, *p* < 0.0001 on day 14), IL-10 (day 0 and 7, *p* < 0.05), and IL-12 (day 14, *p* < 0.006) concentrations. There were no significant differences in KC levels. Unpaired Student’s *t*-test; data are presented as mean, error bars: SD; the total number of samples: *n* = 65, total number per variant at each time point: *n* = 3–5. The total number of biological replicates per group: *n* = 5. Abbreviations: CSF-cerebrospinal fluid.

In Figure 9, IL-1α, IL-10, IL-12, and KC levels in serum (days: 0, 7, 14, 28 post-transplantation) and CSF samples (days: 0, 7, 14 post-transplantation) for the allogeneic C57BL/6 mouse model are presented. The lowest IL-1α level in the C57BL/6 allogenic model was detected in serum in variants in which Tacrolimus and co-stimulatory blockade were applied. Interestingly, on day 14, the IL-1α level decreased in variants in which PiF (46.41 pg/mL) was applied, and this was comparable to the levels obtained for Tacrolimus (41.89 pg/mL) and co-stimulatory blockade groups (45.52 pg/mL). The highest overall IL-1α level was found in the group with no immunosuppression applied, and the highest concentrations were obtained on day 14 post-transplantation (124.04 pg/mL, one-way ANOVA *p* < 0.0001). There were no significant differences in concentration levels of IL-1α in CSF samples; however, overall concentrations decreased with the observation time, except for the co-stimulatory blockade variant, in which concentrations between days 14 and 28 were similar. The lowest level of IL-10 was found in the serum in the variant where no immunosuppression was exerted (17.12–27.35 pg/mL) and the variant with Tacrolimus (22.08–39.70 pg/mL). The highest level of anti-inflammatory IL-10 was detected in serum in PiF (33.93–39.60 pg/mL) and co-stimulatory blockade variants (27.33–44.59 pg/mL). The overall concentration of IL-10 was the highest in the co-stimulatory blockade variant and increased with time post-transplantation. Significant differences were observed on days 0 and 7 in IL-10 serum concentrations between variants applied (one-way ANOVA, *p* < 0.05). There were no significant differences calculated for IL-10 concentration in CSF samples at any time point; however, the highest concentration, in general, was found in a co-stimulatory blockade group (91.18–215.51 pg/mL) and the lowest in the Tacrolimus group (12.71–91.57 pg/mL). The concentration of IL-12 in serum was rather stable when compared between variants applied at day 0 and 7 post-transplantation; however, at day 14 and 28 post-transplantation, there were statistically significant differences (one-way ANOVA *p* < 0.006 and *p* < 0.05, respectively). The lowest concentration of IL-12 in serum was detected in Tacrolimus and co-stimulatory blockade variants at day 14 (316.99 pg/mL and 343.52 pg/mL, respectively) and the highest in the PiF variant (455.98 pg/mL). IL-12 level on day 28 in the Tacrolimus group increased up to 482.63 pg/mL. In CSF samples, the concentration of IL-12 was found to be increased at days 7 and 14 post-transplantation and was the highest in the group with no immunosuppression applied (1297.36–1723.86 pg/mL). There were no significant differences in KC levels in serum between the variants under study; however, the highest level could be observed on days 0 and 7 in the PiF variant and days 14 and 28 in the co-stimulatory blockade variant. In the CSF samples on day 7 post-transplantation, KC level increased in the immunosuppression-free group and co-stimulatory blockade variant and was just slightly changed in the PiF and Tacrolimus variants (mice groups). KC levels increased on day 14 with no immunosuppression, PiF, and Tacrolimus variants; however, no significant differences were found.

### 3.9. Post-Mortem Evaluation of GRPs’ Graft Localization

**Figure 10:**
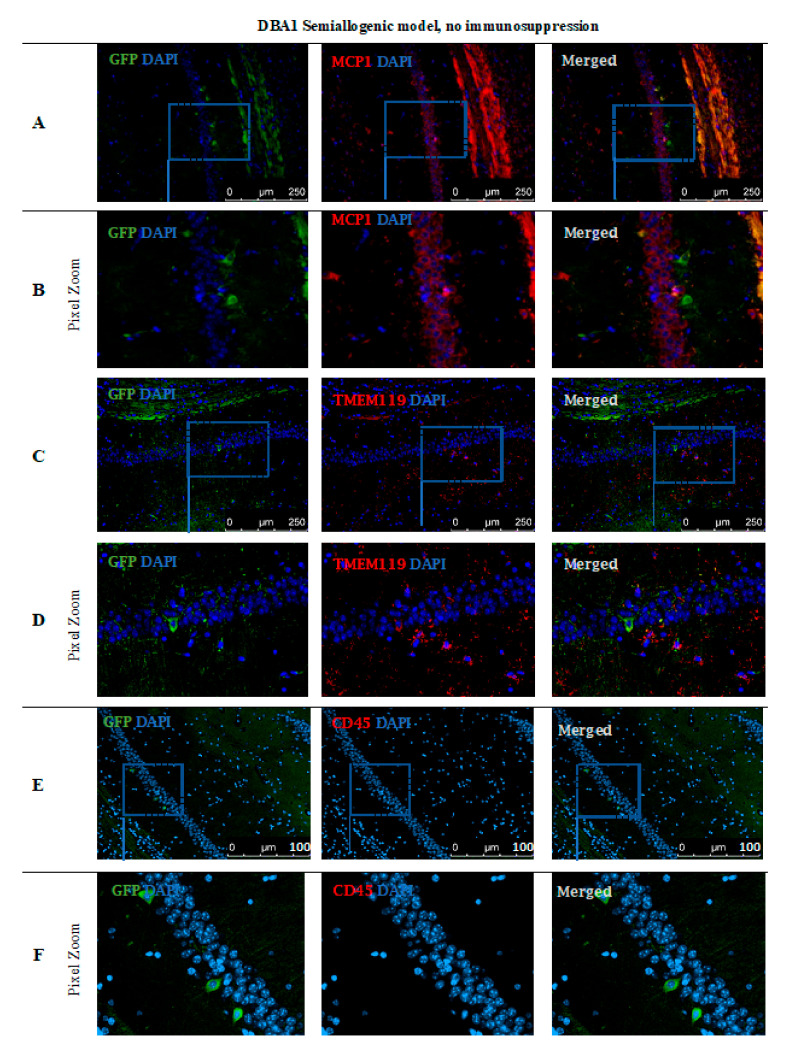
Post-mortem evaluation of GRPs’ graft localization in DBA1 semiallogenic model without immunosuppression applied: Immunohistochemical staining in the brain of DBA1 (semiallogenic) mice after 140 days of observation in a group with no immunosuppression applied. The staining revealed murine GRP GFP+ cells residing in the hippocampus, mostly in regio superior. Grafted cells were present close to MCP-1+ immune cells (**A**,**B**), as well as microglial cells (TMEM119+) (**C**,**D**). No CD45+ cells were visible in the adjacent tissues (**E**,**F**). Abbreviations: GFP-green fluorescent protein; GRP-glial-restricted progenitor cell.

Immunohistochemical staining (Figure 10) performed in the DBA1 semiallogenic mouse model after 140 days of observation confirmed the results performed with the BLI method. In the brains of animals that accepted the GRPs’ graft, numerous cells positive for staining with anti-GFP antibody (green) were found. The GFP staining was performed because of the risk of losing fluorescent signal after all tissue preparation procedures. Additional stainings for the presence of immunocompetent cells of an innate immune response, like microglial cells (TMEM119+) or the presence of the MCP-1, were also performed. The GFP+ cells were present in the whole hippocampus, but mostly in regio superior in the semiallogenic DBA1 mouse model. They were localized in the niche close to activated TMEM119+ microglial cells (Figure 10C,D) as well as MCP-1 positive cells (Figure 10A,B). This observation suggests that TMEM119+ and MCP-1+ cells did not struggle with the transplanted GRPs. No CD45+ cells were present in the area (Figure 10E,F).

**Figure 11:**
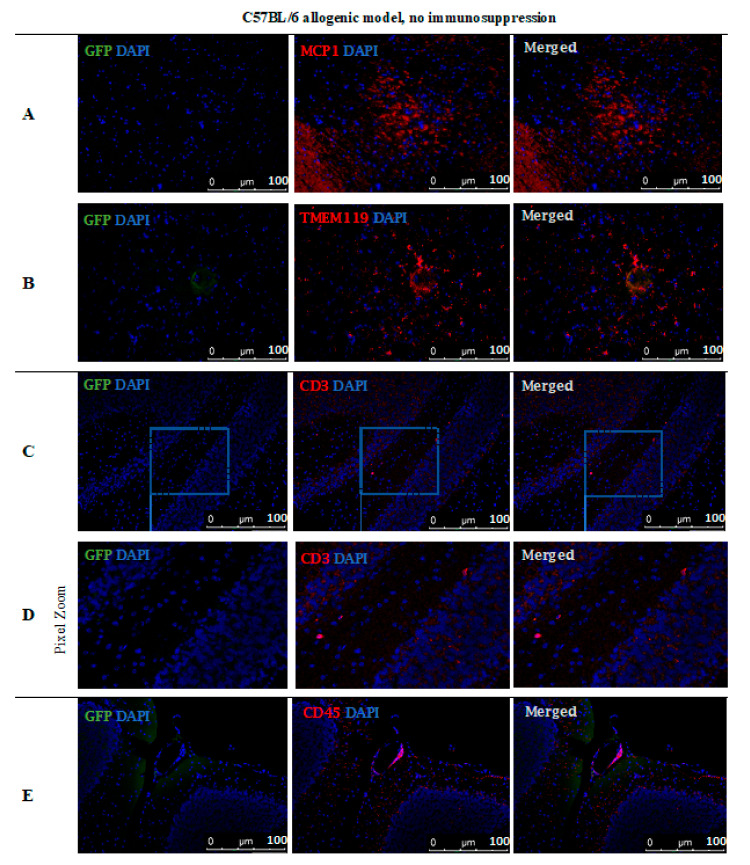
Post-mortem evaluation of GRPs’ graft localization in C57BL/6 allogenic model without immunosuppression applied: Immunohistochemical staining in the brain of C57BL/6 (allogeneic) mice after 14 days of observation in a group with no immunosuppression applied. The staining revealed the presence of MCP-1 in the tissue (**A**). Singular GFP+ cells aggregates were observed in tissue parenchyma surrounded by TMEM119+ microglia (**B**). The bottom picture shows infiltration of CD45+ cells into artery epithelium (**E**). Singular CD3+ cells were also present in brain parenchyma, mostly in the cerebellum, brainstem, and medulla oblongata areas (**C**,**D**). Abbreviations: GFP-green fluorescent protein; GRP-glial-restricted progenitor cell.

However, no GFP-positive cells were observed after 14 days post-transplantation in the allogeneic C57BL/6 mouse model with no immunosuppression applied. The staining revealed the presence of MCP-1 in the tissue (Figure 11A) and TMEM119+ microglia cells (Figure 11B). Singular CD3+ (Figure 11C,D) and CD45+ (Figure 11E) immune cells were present, infiltrating brain tissues and brain blood vessel adjacent areas (Figure 11).

**Figure 12:**
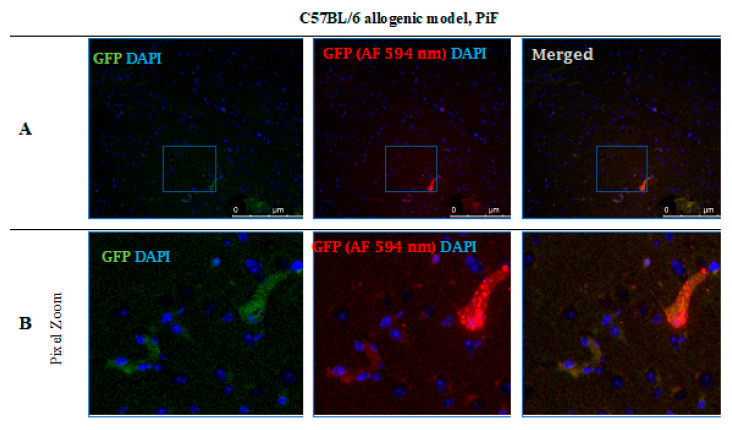
Post-mortem evaluation of GRPs’ graft localization. Immunohistochemical staining in the brain of C57BL/6 (allogeneic) mouse model after 14 days of observation in the group where PiF was applied: The GFP+ cells (green) were observed in the midbrain tissue. We used an anti-GFP antibody (secondary antibody pertaining to red fluorescence Alexa Fluor 594 nm, Abcam, Cambridge, UK) to assess if the signal is specific (**A**,**B**). Abbreviations: GFP-green fluorescent protein; GRP-glial-restricted progenitor cell; PiF-preimplantation factor.

After 14 days post-transplantation in the PiF group, the staining revealed single GFP positive cells present in the midbrain parenchyma (Figure 12).

### 3.10. Graft Survival in ALS-SOD1G93A Model

**Figure 13:**
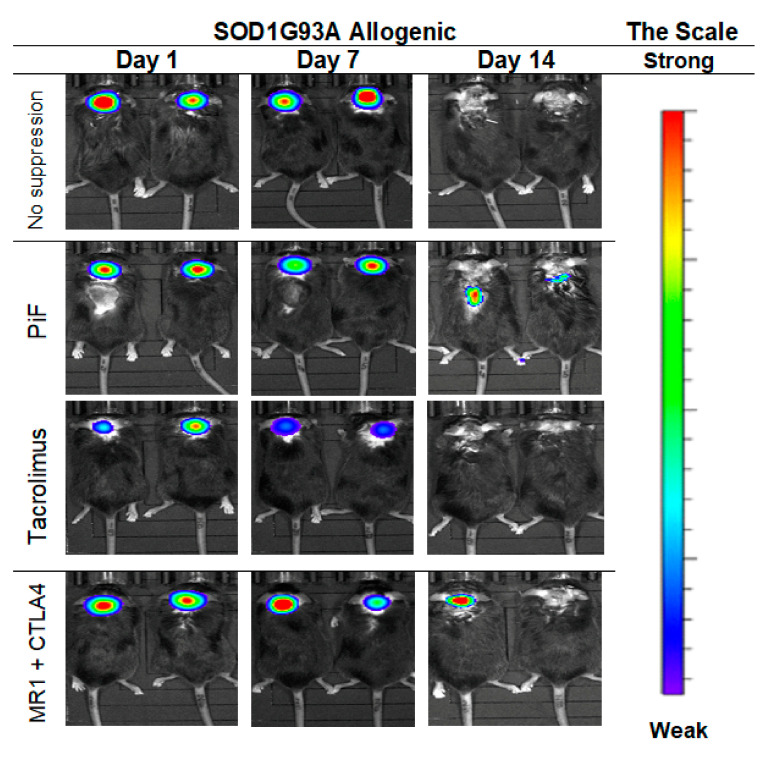
Murine GRP’s survival after cell transplantation in ALS- SOD1G93A allogenic model: The picture represents the observation performed on days 1, 7, and 14. The best visible signal on day 14 was detected in PiF and co-stimulatory blockade variants. Abbreviations: ALS-amyotrophic lateral sclerosis; GRP-glial-restricted progenitor cell; PiF-preimplantation factor.

The cellular graft containing GRPs was transplanted into 10-week old male SODG93A mice with immunosuppressive or immunomodulatory protocols introduced as described previously with or without immunosuppression. The graft was visible from day 1 to day 7 in the group with no immunosuppression, but the signal disappeared on day 14 from GRPs’ delivery. Similar results were seen after PiF application; however, the visible signal on day 14 in a spine region was still detected. Graft survival was poorly visible in the Tacrolimus variant, in which the BLI signal was weaker on day 7 as compared with the other groups and not detectable on day 14. Satisfactory GRPs’ graft survival was observed in the variant in which co-stimulatory blockade was applied (Figure 13).

### 3.11. Cytokine Levels in Serum and CSF Samples in ALS SOD1G93A Allogenic Mouse Model

**Figure 14:**
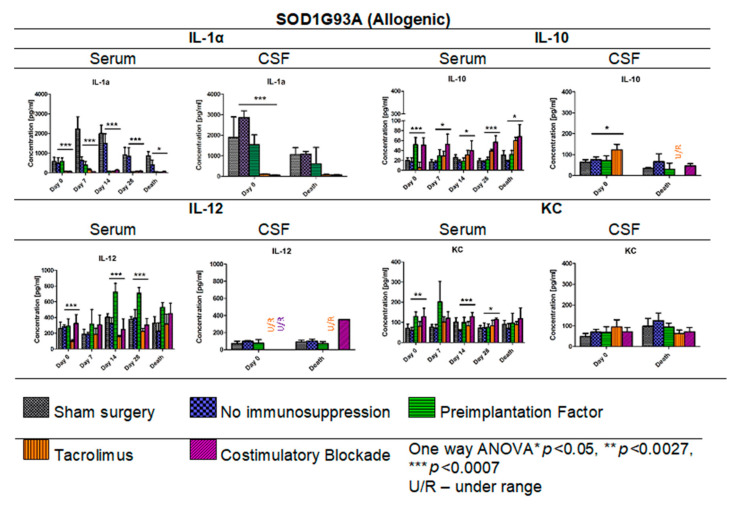
Cytokine levels in serum and CSF samples in ALS SOD1G93A allogenic mouse model: IL-1α, IL-10, IL-12, and KC levels in serum and CSF samples were measured in allogenic ALS SOD1G93A mice. There was a striking difference in IL-1α levels in no immunosuppression and sham surgery variants and variants in which immunosuppressive versus immunomodulatory regimens were applied. Significant differences were also seen in IL-10, IL-12, and KC levels in serum samples and IL-1α and IL-10 levels in CSF samples on day 0. No significant differences were found in IL-12 and KC levels in the course of observations. Moreover, no significant differences on the day of termination for IL-1α and IL-10 levels were found. However, it is worth emphasizing that the levels of IL-10 and IL-12 in the Tacrolimus and co-stimulatory groups were undetectable in CSF. Information about other cytokines levels from the 23-cytokine mouse panel is accessible in Supplementary Figures S1–S3. Unpaired Student’s *t*-test; data are presented as mean, error bars: SD; the total number of samples: *n* = 101, total number per variant at each time point: *n* = 2–6. The total number of biological replicates per group: *n* = 6. Abbreviations: ALS-amyotrophic lateral sclerosis; CSF-cerebrospinal fluid.

In Figure 14, the results of Multiplex ELISA analysis for IL-1α, IL-10, IL-12, and KC cytokine levels in ALS SOD1G93A mouse allogenic model in serum (days: 0, 7, 14, 28, and the day of termination) and CSF samples (day 0 and the day of termination) are presented. The highest concentration in general for IL-1α was observed in the variant in which no immunosuppression was applied (408.19–1501.60 pg/mL) and the lowest in Tacrolimus (32.91–150.27 pg/mL) and/or co-stimulatory blockade (60.39–130.21 pg/mL) variants. The IL-1α levels in the PiF variant decreased after cell transplant administration from 582.19 pg/mL at day 0 to 47.09 pg/mL on day 28. Interestingly, there were no significant differences observed in IL-1α concentrations on day 7 between sham surgery (2639.39 pg/mL) and the variant in which no immunosuppression was applied (2631.89 pg/mL), suggesting that GRP graft may have itself anti-inflammatory properties. On day 0, the lowest level of IL-1α was detected in the variants in which Tacrolimus or co-stimulatory blockade were applied, but the highest was observed in the variant with no immunosuppression. On the day of mouse termination, overall IL-1α levels were low in all the groups; however, the lowest levels were observed for Tacrolimus and co-stimulatory blockade variants. At each time point, significant differences were observed between the variants applied; one-way ANOVA *p* < 0.0007 on days 0, 7, 14, and 28, and *p* < 0.05 on the day of animal termination. The highest levels of IL-1α in CSF samples were detected on day 0 for no immunosuppression groups (2855.01 pg/mL) and the PiF (1539.82 pg/mL) variant, but the lowest for the Tacrolimus (102.77 pg/mL) and co-stimulatory blockade (53.05 pg/mL) variants. At this time point, significant differences between applied variants for IL-1α CSF concentration (one-way ANOVA *p* < 0.0007) were observed. There were significant differences noted in the concentrations of IL-10 in serum-the highest one was observed on day 0 for PiF and co-stimulatory blockade (51.55 pg/mL and 50.87 pg/mL, respectively) variants and the lowest for the Tacrolimus variant (5.40 pg/mL); however, this trend changed with time-IL-10 serum levels increased in the Tacrolimus and co-stimulatory blockade variants, whereas for PiF and no immunosuppression groups, they were relatively stable. Variant differences in IL-10 concentration in serum resulted in a significance at each time point, *p* < 0.0007 for days 0 and 28, and *p* < 0.005 for days 7, 14, and the time of termination. The differences in IL-10 level were also found in the CSF samples at the starting point (day 0, *p* < 0.005 one-way ANOVA), and the highest was in the Tacrolimus group (122.74 pg/mL). The level of IL-10 in CSF samples decreased and was very low or undetectable on the day of mice termination. The lowest serum level of IL-12 was assessed in the Tacrolimus group in all-time observatory points (97.52–318.69 pg/mL); however, on the day of termination, the average IL-12 level in the Tacrolimus group was highest and similar to the sham or no immunosuppression group. The highest IL-12 concentration was found in the serum of the PiF group on day 14 (722.72 pg/mL) and 28 (709.34 pg/mL) post-transplantation. The IL-12 levels in CSF, however, were very low or even undetectable for all groups. The KC serum level was detected as the highest at the day 0 in co-stimulatory blockade (128.52 pg/mL) and PiF (129.43 pg/mL) variants, and the lowest and comparable levels were observed in the no immunosuppression (58.21–73.99 pg/mL) and Tacrolimus (80.70–101.75 pg/mL) groups. There was a visible peak on day 7 post-transplantation in the PiF group; however, the difference was not significant. The KC level in CSF samples was comparable during observation time; however, the highest one was detected at the termination time point in the group with no immunosuppression (124.19 pg/mL), and the lowest one was found in the co-stimulatory blockade variant (71.46 pg/mL). However, no statistical significance was observed.

### 3.12. GRP Cells Localization Post-Mortem in ALS SOD1G93A Mouse Model

**Figure 15:**
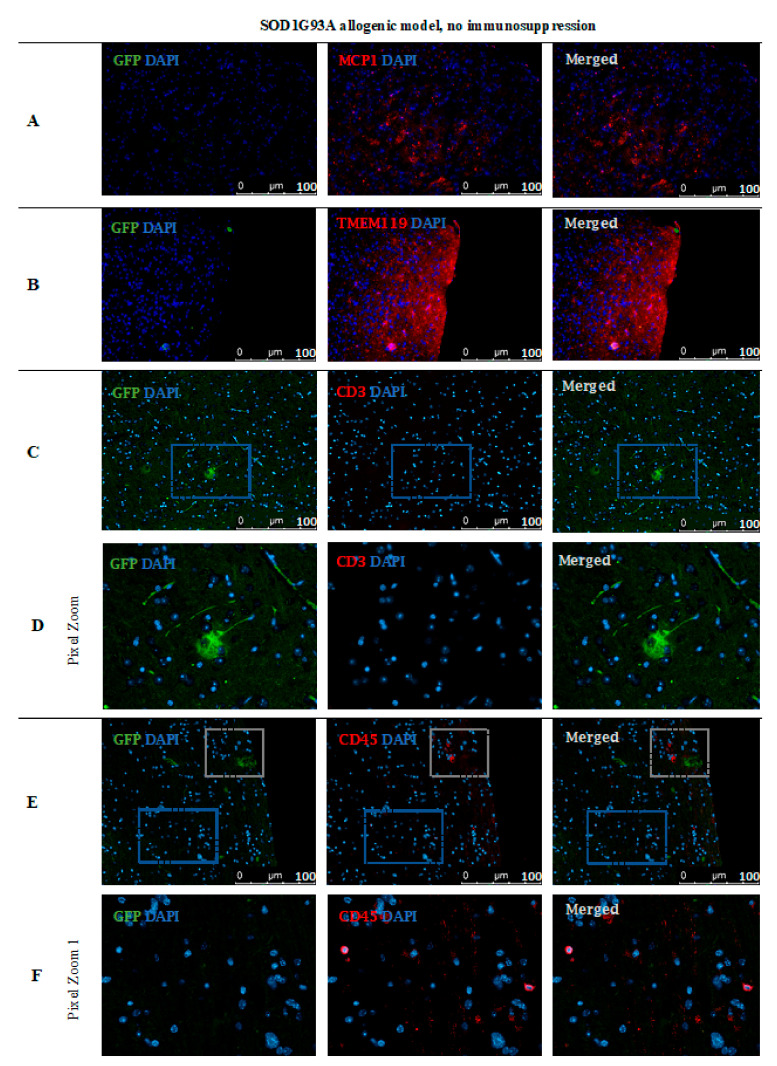
Cell localization post-mortem in ALS SOD1G93A mouse model without immunosuppression applied: Immunohistochemical staining in ALS SOD1G93A (allogenic) mice after 56 days of observation in the group with no immunosuppression applied. There were elevated MCP-1 levels (**A**) in the tissue, TMEM119 (**B**) and CD3+ (**C**,**D**), suggesting microglia activation. The GFP+ cells were very rare and the medium number of CD45+ cells with lymphocyte and macrophage morphology was present in the brain tissue (**E**,**F**). Abbreviations: ALS-amyotrophic lateral sclerosis; GFP-green fluorescent protein.

Based on immunofluorescence staining in ALS SOD1G93A mice, the disappearance of transplanted GRP cells was confirmed after 56 days post-transplantation in the immunosuppression-free group (Figure 15). Single GFP+ cells were visible in the brain parenchyma; however, immune cell infiltration was also observed, which indicates ongoing inflammation in the tissue. As presented in Figure 15A, there was visible elevated MCP-1+ cell expression in brain tissue as well microglia activation, which was confirmed by expression of TMEM 119+ (Figure 15B). Infiltration of scattered CD45+ T cells was observed-mostly in the area of cerebellum and brainstem and *medulla oblongata* (Figure 15E–G).

## 4. Discussion

Glial-restricted progenitors are considered a promising tool for cellular therapy in ALS because, under proper conditions, they can differentiate into astrocytes and oligodendrocytes and may contribute to the repair of damaged neurons. However, the regenerative potential of GRPs is restricted by the route of cells’ delivery and poor graft survival in allogeneic conditions. Previous studies documented that GRPs, when transplanted into the central nervous system (CNS) under cover of mesenchymal stem cells of bone marrow-origin (BM-MSC), can generate a pro-regenerative effect in the spinal cord injury in a rat model [22]. In the preclinical ALS SOD1G93A mouse model, several strategies for the best source of stem/progenitor cells and delivery routes have been tested. The most promising beneficial effect on delayed disease onset and amelioration of motor neuron death was observed after intrathecal BM-MSCs cells’ delivery (reviewed by Ciervo Y et al. and Thomasen et al.) [23,24].

In this study, we present an attempt to assess the best strategy for GRPs’ grafting protocol, directly administering cells into CNS, intrathecally into cisternum magnum with or without applied immunomodulation/immunosuppression. We tried to answer the main question: “whether an application of immunosuppression is necessary when considering GRP transplanting into CNS?”. To answer this question, we selected three mouse models: semiallogeneic (DBA1), allogeneic: healthy mice (C57BL/6), and ALS SOD1 G93A-disease mice. The GRPs obtained from fetal tissues were collected and banked for therapeutic application. The substantial aspect of the study was an immunological background of interplay assessment between the graft [18] and mouse immunity (systemic and local), and the assessment of the route of graft delivery by MRI with required skills to exert the lowest possible risk of neural tissue damage.

The main limitation of the study was some inconsistency in continuous observation of graft survival by BLI. In particular, for C67BL/6 PiF and no immunosuppression variants (groups), some observations took place just for 14 days because of the necessity to collect the tissue for histological analysis. This limitation was mainly related to the necessity to lower the number of mice used in the experiment owing to ethical regulations. The observation was stopped when the BLI signal was not detectable in two consecutive measurements, and animals were then terminated. However, the subsequent immunohistochemical assessment revealed the presence of GFPs+ cells in the brain parenchyma. As might be expected, cell numbers varied in all the combinations studied and were not displayed in high numbers, including the semiallogeneic combination, which has been the longest one in terms of in situ incubation. Indeed, it was interesting to see whether bioluminescent signal loss correlated with either GRPs’ migration/retention or rather immune-mediated cell presence (allogeneic combination). The adavantage of semiallogeneic GRPs’ survival does not automatically give them an advantage over the other sytems studied with respect to their functionality and disease amelioration. This issue is now intensively studied in our laboratory, including behavioral mouse monitoring systems.

There is no simple answer as to whether co-administration of immunosuppressive/immunomodulatory factors is necessary when GRPs are transplanted into cisterna magna. In the semiallogeneic model, with halfway histocompatibility matched GRPs, the graft survival was superior without any additional regimen applied. The CNS may constitute an immune-privileged compartment, but in certain conditions, lymphocytes may cross the blood–brain barrier (BBB) and infiltrate brain tissue, as the cells can be previously activated in lymph nodes by dendritic cells. The brain danger or damage associated molecular patterns (PAMPs and DAMPs) mean that APCs present inside peripheral lymphatic organs are drained from the brain parenchyma into the lymphatic system and may travel into lymph nodes where they are involved in T-cells’ activation. Activated T-cells might then enter the peripheral blood system and travel through CNS [13]. A fascinating theory about the mechanisms of CNS infiltration by T-cells and the role of microglial cells was reviewed and explained by Schetters and Gomez-Nicola et al. [13]. They suggested that microglia actually might play a more immunosuppressive and pro-regenerative role inside the CNS and are able to minimize the damage related to the inflammatory response of T-cell infiltration. A similar phenomenon was observed in our study when GRP administration in semiallogenic DBA1 model (illustrated in the pictures presenting graft survival at the day 140 post-transplantation) (Figure 10) showed that the GFP+ cells were living in hippocampus in a close proximity of TMEM-119+ microglia cells, as well as in neighborhood MCP-1+ cells co-existing without peripheral leukocyte infiltration.

Therefore, one can speculate about the potential role of the undisrupted cellular niche (including microglia) for better graft survival. In this line, we decided to present IHC analysis in mice in which no immunosuppression was applied in conditions not influenced by external immunomodulatory factors. This approach illustrated well what was happening inside the CNS after GRPs’ graft administration. Experiments with grafts of different degrees of histocompatibility provided proof that semi-allogenic histocompatibility matching may be sufficient for graft survival when considering GRP cell administration to CNS.

In studies on the possible therapeutic effect of fetal embryonic cortical tissue transplanted into the host brain parenchyma, it was reported that the microglial cells residing in the fetal tissue vanished quickly, and host-derived microglia cells inhabited transplanted neural tissue without any harm to it. Thus, the microglia cells play an essential role in CNS homeostasis-not only as immunocompetent cells, but also as regulators of the neural network after fetal development and rearrangement while learning their function in late ontogeny [25].

In the DBA1 mouse semiallogenic model, immunosuppressive factors that inhibit the immunological response appear to make neuroprotective features of immune cells within the CNS redundant. Interestingly, in the semiallogenic DBA1 mouse model, in the immunosuppression-free group, the IL-1α level in the peripheral blood on day 7 was relatively low, but significantly increased on day 14. On this day, the BLI signal from the graft was the lowest, but the signal strength improved in the next week. Similar IL-1α upregulation was observed in the peripheral blood of the allogeneic C57BL/6 model; however, no improvement in the graft survival was noticed in the immunosuppression-free group. Application of immunosuppression decreased IL-12 levels in the blood of C57BL/6 allogenic mice and in semiallogenic DBA1 mice on day 14.

Interestingly, in both models, on day 14, IL-12 expression in CSF was visibly higher when no immunosuppression was applied. IL-12 in CNS is produced by activated microglial cells and increased after GRPs’ transplantation as a natural response-possibly in order to home the immune cells [26]. In both mouse models, the best survivals were seen in variants in which IL-10 expression in CSF was the highest, and this was related to immunosuppression-free and PiF variants in the semiallogenic DBA1 mouse model and co-stimulatory blockade variant in the allogeneic C57BL/6 model.

KC (CXCL1) is a chemokine protein that plays a pleiotropic role inside the CNS-it might act as a chemoattractant for neutrophils, but it was also proven to have immunoprotective properties in multiple sclerosis [27]. We did not see any significant changes in its concentration within CNS after GRP grafting; however, its levels in serum of co-stimulatory molecules blockade variant of DBA1 mouse semiallogenic model were generally higher. Our previous work has shown that subpopulations of murine GRPs express co-stimulatory molecule CD40, and this feature was not observed in human nor canine cells [18]. Taking this together with the fact of TLR-4 expression on the mouse GRPs, there is the possibility that the GRPs might be able to express co-stimulatory molecules and MHC-II after IFN-γ, LPS, or HSP-70 stimulation [28,29]. This possibility should be further studied to better understand the biology and immunological potential of the GRPs. This hypothesis could match our observations that, in the semiallogenic model, GRPs survived well without application of any immunosuppression. As CNS can be treated as an immune-privileged site, only limited infiltration of T-cells can be possible; thus, better histocompatibility matching could convert local microglia into M2-like protective subtypes. This hypothesis is quite possible, because, in the DBA1 semiallogenic model where no immunosuppression was applied, the highest overall concentrations of IL-13 were observed, which is known to be a factor that influences microglia to enter its M2-like subtype (own data available upon the request) [30,31].

The IL-1α levels in ALS SOD1 G93A allogenic mouse model were much higher in sham and no immunosuppression variants (in serum and CSF samples) compared with mouse groups with exerted immunosuppression/immunomodulatory regime, suggesting a high pro-inflammatory environment. However, GRPs graft alone was sufficient to put down IL-1α levels in the peripheral blood compared with the levels observed in the sham surgery mouse group. The neuroinflammation within CNS in ALS mice might cause increased infiltration of immunocompetent cells owing to tissue degeneration and BBB disruption [32], affecting graft survival. In contrast, the level of IL-12 in the ALS SOD1 G93A allogenic mouse model was the lowest in the Tacrolimus variant and highest in the PiF group, and this observation is different when compared with DBA1 semiallogenic and C57BL/6 allogenic models. This unfavorable environment in ALS could be the reason for the very poor graft survival noted there, which led us to attempt to answer the second question-whether GRPs grafting in ALS SOD1 G93A mouse model can have practical therapeutic meaning? Unfortunately, we did not observe significant differences in overall mice survival; however, single mice after GRP transplantation did perform better. It could be too early for the reason for these disparate observations to be explained by cell grafting alone-as we applied them before the first ALS symptoms. It is worth emphasizing that, in the allogenic model, the graft survived poorly (compared with semiallogenic model) regardless of applied immunosuppression/immunomodulation. Weeks after transplantation, when neurodegeneration in the SOD1 mouse model was progressing, there were most probably no GRP cells left to repair this damage. Thus, the graft survival in the allogenic ALS SOD1G93A model behaved even poorer than in the allogeneic C57BL/6 mouse model. Therefore, the protocol of using GRP to treat ALS symptoms in the SOD1G93A mouse model should be further optimized.

In summary, the results may suggest the importance of histocompatibility matching and its potential role of microglia in maintaining of GRPs’ graft survival. As microglia are immune cells responsible for maintaining the pro-regenerative environment and proper neural network development, the influence of immunosuppressive factors might actually exert a deteriorating effect on its biology, particularly in semiallogenic conditions when the graft seems to have the potential to survive itself. In such a scenario, the application of immunosuppression regime should be considered case-dependently and should be based on a previous assessment of histocompatibility between donor cells and recipient. It should also be remembered that there is also a necessity to reconsider the animal model for ALS-GRP transplantation studies as differences in the expression of co-stimulatory molecules in murine GRP versus other species may lead to tremendous differences in immunological response.

## Figures and Tables

**Table 1:** Antibody characteristics.

**Immunofluorescent Staining**					
**Marker**	**Isotype**	**Company**	**Reactivity**		**Dilution**
Nestin	IgG I	Abcam, Cambridge, UK	M		1:20
GFAP	IgG	Abcam, Cambridge, UK	Ma		1:1000
PSA-NCAM	IgM	Merck, Darmstadt, Germany	Ma		1:200
A2B5	IgM	Merck, Darmstadt, Germany	Ma		1:30
NG2	IgG I	Abcam Cambridge, UK	M		1:100
**Flow Cytometry**					
**Marker**	**Isotype**	**Company**	**Reactivity**	**Fluorochrome**	**Dilution**
GFAP	IgG2b κ	BD Biosciences, San Jose, CA, USA	Ma	AF 647 nm	1:25
NG2	IgG I	Abcam, Cambridge, UK	M, Hu	-	1:20
Mouse IgG I κ, Isotype control	IgG I κ control	Abcam, Cambridge, UK	-	-	1:50
Mouse IgG2b κ, Isotype control	IgG2b κ control	BD Biosciences, San Jose, CA, USA	-	AF 647 nm	1:10
A2B5	IgM	Miltenyi Biotec, Bergisch Gladbach, Germany	M, Hu	APC	1:20
PSA-NCAM	IgM	Miltenyi Biotec, Bergisch Gladbach, Germany	Ma	APC	1:20
Mouse IgM, Isotype control	IgM control	Miltenyi Biotec, Bergisch Gladbach, Germany	-	APC	1:20

Hu: human; M: murine; Ma: mammals; AF: Alexa Fluor; Nestin: neural stem cell marker; PSA-NCAM: polysialylated–neural cell adhesion molecule (marker of neural precursor cells); A2B5: marker of neural progenitor cells; GRP cells: type II astrocytes; GFAP: glial fibrillary acidic protein (astrocytemarker, glial cell marker); NG2: nerve/glial antigen 2 (marker of oligodendrocyte precursor cells (OPCs).

## Supplementary Materials

The following are available online at https://www.mdpi.com/article/10.3390/cells10071804/s1, Figure S1: The picture presents multiplex ELISA in serum and CSF of C57BL6 mice in various immunomodulatory and immunosuppressive regimens. Two-way ANOVA; error bars: SEM; *n* = 3–6 samples per experimental group. Sham group was excluded from statistic calculations. Figure S2: The picture presents multiplex ELISA in serum and CSF of DBA1 mice in various immunomodulatory and immunosuppressive regimens. Two-way ANOVA; error bars: SEM; *n* = 2–6 samples per experimental group. Sham group was excluded from statistic calculations. Figure S3: The picture presents multiplex ELISA in serum and CSF of SOD1 G93A mice in various immunomodulatory and immunosuppressive regimens. Two-way ANOVA; error bars: SEM; *n* = 2–6 samples per experimental group. Sham group was excluded from statistic calculations.
