# Supplementary material for: Assessment of Immunological Potential of Glial Restricted Progenitor Graft In Vivo—Is Immunosuppression Mandatory?
Source: Cells. 2021 Jul 16;10(7):1804. doi: 10.3390/cells10071804 (PMC8308088; doi:10.3390/cells10071804)
Supplement: Supplementary file 1 [file cells-10-01804-s001.zip › cells-1258389-supplementary.pdf]

Supplementary Figure:

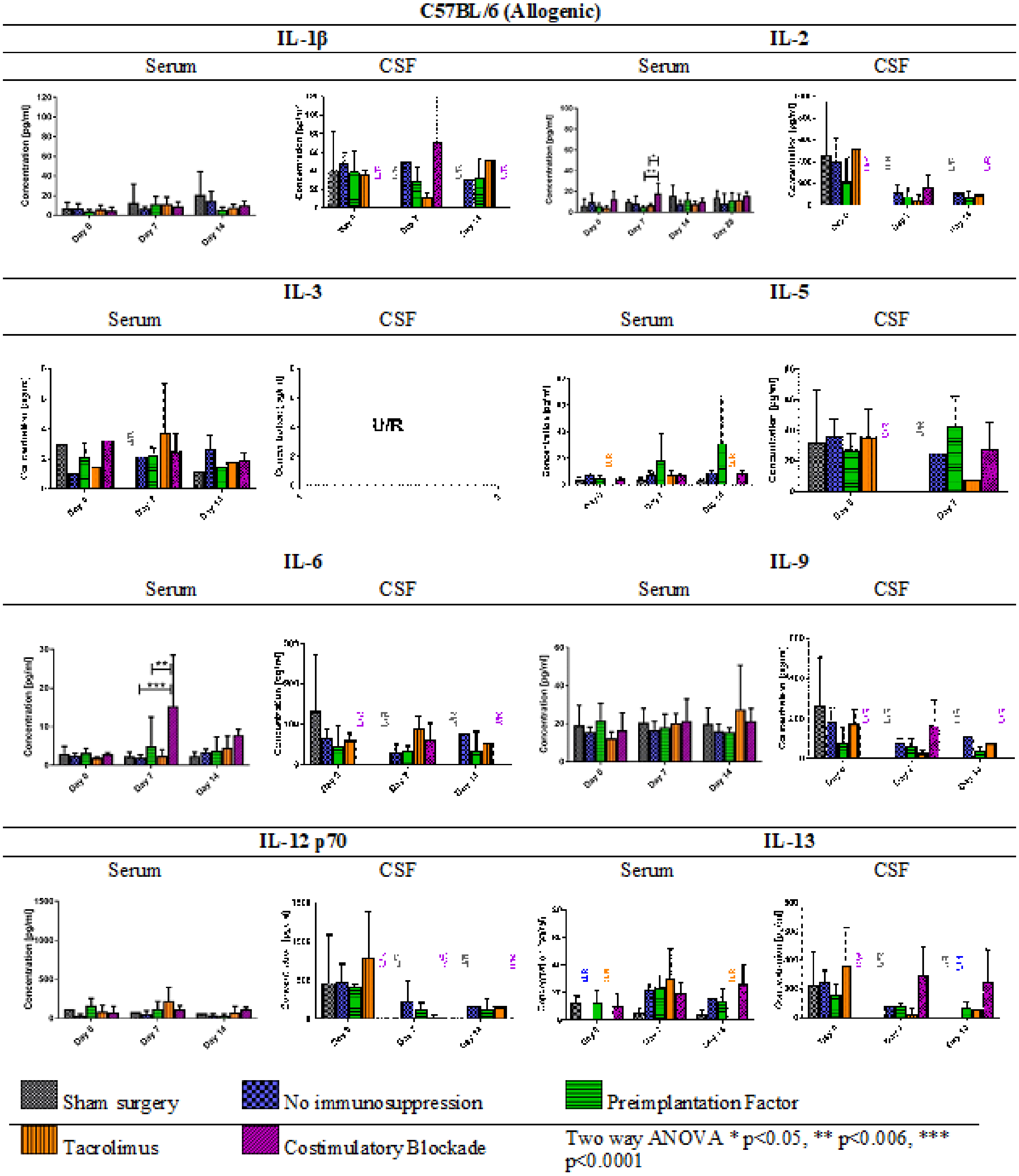

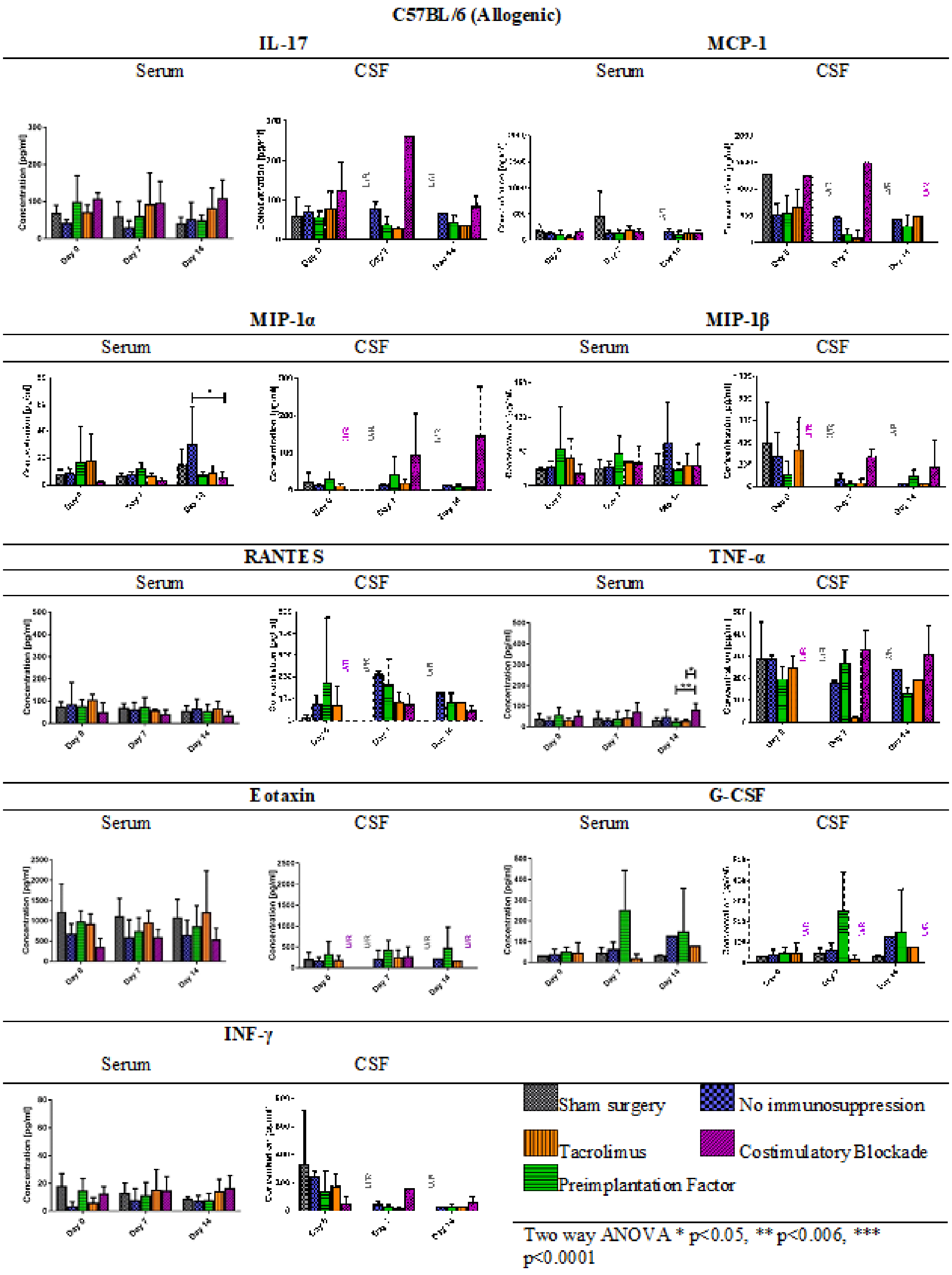

Fig.S1. The picture presents multiplex ELISA in serum and CSF of C57BL6 mice in various immunomodulatory and immunosuppressive regimens. Two-way ANOVA; error bars: SEM; n = 3-6 samples per experimental group. Sham group was excluded from statistic calculations.

IL-1β: there is no significant difference in IL-1β levels between groups (serum and CSF),

IL-2: there is significant difference in serum IL-2 levels between:

PiF [12.98 pg/mL] and costimulatory blockade [35.05 pg/mL] (** $p<0.01$),

tacrolimus and costimulatory blockade (* $p<0.05$) on day7.

No significant difference measured in CSF,

IL-3: there is no significant difference between groups in IL-3 (serum). In CSF the measurements were under detectable range.

IL-5: there is no significant difference in IL-5 levels between groups (serum and CSF).

IL-6: there is significant difference on day 7 in serum IL-6 levels between:

no immunosuppression group [4.44 pg/mL] and costimulatory blockade group [13.30 pg/mL] (*** $p<0.001$)

and preimplantation factor and costimulatory blockade group (**$p<0.01$).

No significant differenced counted in CSF.

IL-9: there is no significant difference in IL-9 levels between groups (serum and CSF),

IL-12 (p70): there is no significant difference in IL-12 (p70) levels between groups (serum and CSF),

IL-13: there is no significant difference in IL-13 levels between groups (serum and CSF), although IL-13 levels were measured as visibly highest in CSF in costimulatory blockade group on day 7 and day 14,

IL-17: there is no significant difference in IL-17 levels between groups (serum and CSF),

MCP-1: there is no significant difference in MCP-1 levels between groups (serum and CSF),

MIP-1α: there is a significant difference in MIP-1α serum level between no immunosuppression group [30.33 pg/mL] and costimulatory blockade group [6.14 pg/mL] (* $p<0.05$) on day 14. No significant difference measured in CSF,

MIP-1β: there is no significant difference in MIP-1β levels between groups (serum and CSF),

RANTES: there is no significant difference in RANTES levels between groups (serum and CSF),

TNF-α: there is a significant difference on day 14 in TNF-α serum level between:

PiF [27.27 pg/mL] and costimulatory blockade [83.52 pg/mL] (** $p<0.01$),

tacrolimus [30.17 pg/mL] and costimulatory blockade [83.52 pg/mL] (* $p<0.05$).

Eotaxin: there is no significant difference in Eotaxin levels between groups (serum and CSF),

G-CSF: there is no significant difference in G-CSF levels between groups (serum and CSF), however highest concentrations were measured in PiF group, both in serum and CSF.

IFN-γ: there is no significant difference in IFN-γ levels between groups (serum and CSF),

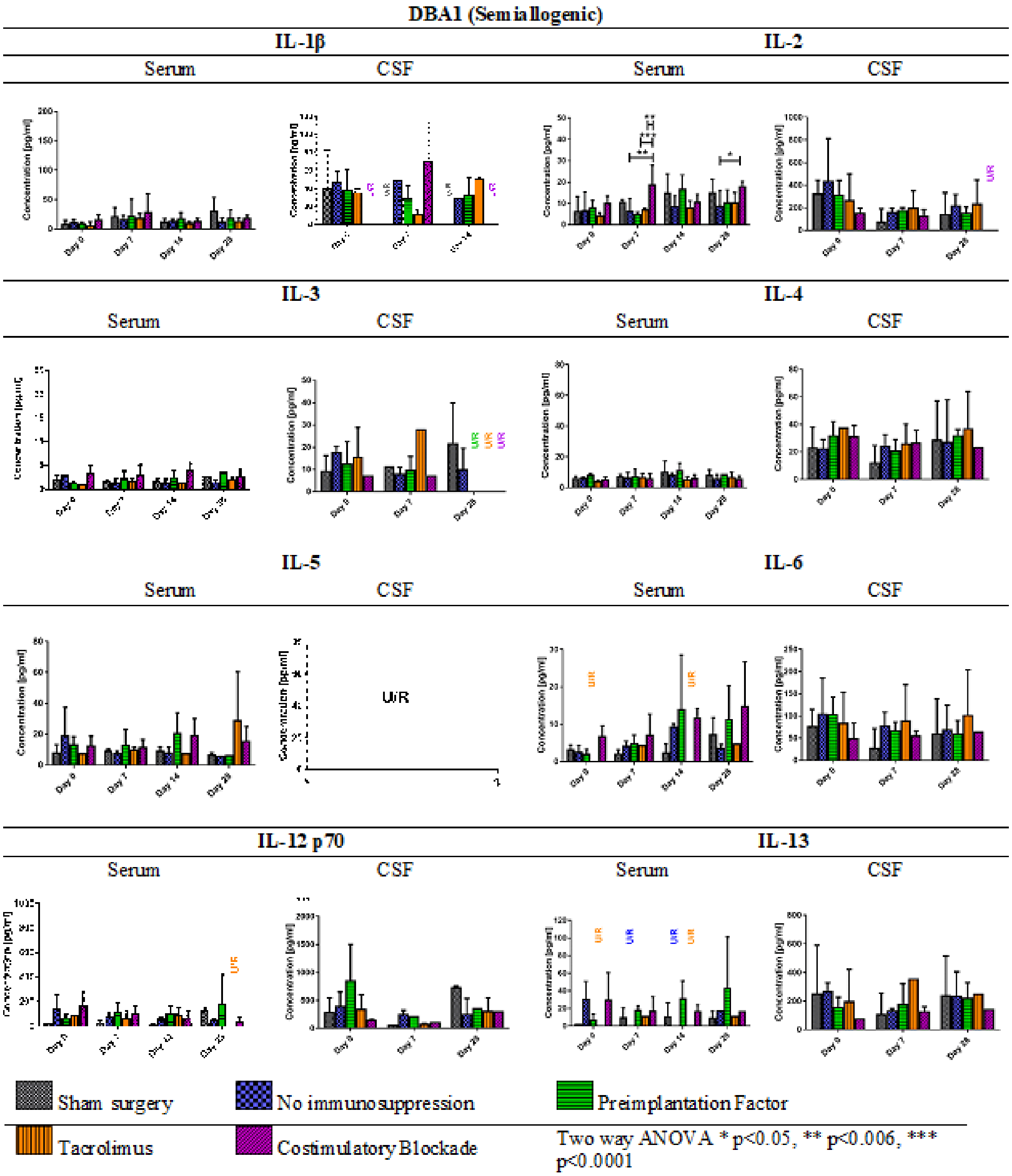
DBA1 (Semiallogenic)
IL-1β
IL-2
Serum
CSF
Serum
CSF
IL-3
IL-4
Serum
CSF
Serum
CSF
IL-5
IL-6
Serum
CSF
Serum
CSF
U/R
IL-12 p70
IL-13
Serum
CSF
Serum
CSF
Concentration [pg/ml]
Day 0
Day 7
Day 14
Day 28
Sham surgery
No immunosuppression
Preimplantation Factor
Tacrolimus
Costimulatory Blockade
Two way ANOVA * p<0.05, ** p<0.006, *** p<0.0001

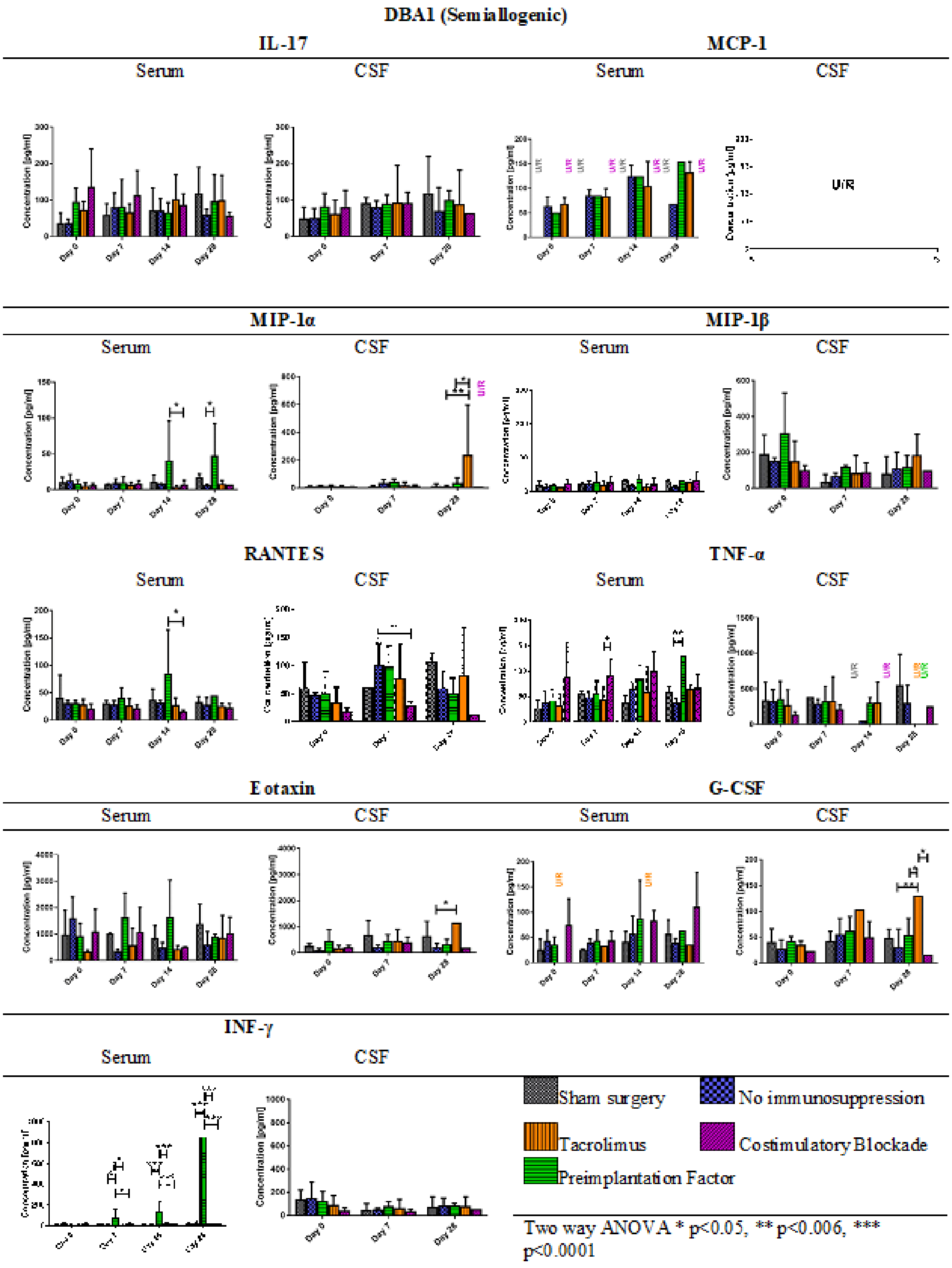

Fig. S2. The picture presents multiplex ELISA in serum and CSF of DBA1 mice in various immunomodulatory and immunosuppressive regimens. Two-way ANOVA; error bars: SEM; n = 2-6 samples per experimental group. Sham group was excluded from statistic calculations.

IL-1β: there is no significant difference in IL-1β levels between groups (serum and CSF),

IL-2: there is significant difference in serum IL-2 levels:

> on day 7 between no immunosuppression [6.51 pg/mL] and costimulatory blockade [18.94] (** $p<0.01$),
>
> PiF [5.01 pg/mL] and costimulatory blockade [18.98 pg/mL] (*** $p<0.001$)
>
> tacrolimus [7.15 pg/mL] and costimulatory blockade [18.98 pg/mL] (** $p<0.01$) groups
>
> on day 28 between no immunosuppression [8.92 pg/mL] and costimulatory blockade [18.17 pg/mL] groups (* $p<0.05$).

No significant difference measured in CSF,

IL-3: there is no significant difference between groups in IL-3 (serum, CSF).

IL-4: there is no significant difference in IL-4 levels between groups (serum and CSF),

IL-5: there is no significant difference in IL-5 levels between groups (serum). In CSF the measurements were under detectable range,

IL-6: there is no significant difference in IL-6 levels between groups (serum and CSF),

IL-9: there is no significant difference in IL-9 levels between groups (serum and CSF),

IL-12 (p70): there is no significant difference in IL-12 (p70) levels between groups (serum and CSF),

IL-13: there is no significant difference in IL-13 levels between groups (serum and CSF),

IL-17: there is no significant difference in IL-17 levels between groups (serum and CSF),

MCP-1: there is no significant difference in MCP-1 levels between groups (serum). In CSF the measurements were under detectable range,

MIP-1α: there is a significant difference in MIP-1α serum level:

> on day 14 between PiF [40.96 pg/mL] and costimulatory blockade group [7.61 pg/mL] (* $p<0.05$)
>
> on day 28 between no immunosuppression group [6.61 pg/mL) and PiF group [47.84 pg/mL] (* $p<0.05$).

The significant differences in MIP-1α in CSF on day 28 was measured between:

> no immunosuppression [11.51 pg/mL] and tacrolimus [237 pg/mL] (** $p<0.01$) group
>
> PiF [37.05 pg/mL] and tacrolimus [237 pg/mL] group (* $p<0.05$).

MIP-1β: there is no significant difference in MIP-1β levels between groups (serum and CSF),

RANTES: there is a significant difference in RANTES levels in serum between PiF [85.25 pg/mL] and costimulatory blockade group [15.59 pg/mL] on day 14 (*$p<0.05$). There was measured significant differences in CSF between no immunosuppression [100.8 pg/mL] and costimulatory blockade group [29.01 pg/mL] on day 7 (* $p<0.05$).

TNF-α: there is a significant difference in TNF-α serum level:

> Day 7 between tacrolimus [44.88 pg/mL] and costimulatory blockade [92.43 pg/mL] (* $p<0.05$)
>
> Day 28 no immunosuppression group [37.97 pg/mL] and PiF [130.7 pg/mL] (** $p<0.01$),

Eotaxin: there is no significant difference in Eotaxin levels between groups (serum). There is significant difference between Eotaxin CSF levels between no immunosuppression group [224 pg/mL] and tacrolimus group [1149 pg/mL] on day 28 (* $p<0.05$).

G-CSF: there is no significant difference in G-CSF levels between groups (serum). There is significant difference in G-CSF levels in CSF on day 28 between:

no immunosuppression [31.62 pg/mL] and tacrolimus group [130.7 pg/mL] (** $p<0.01$),

PiF [54.36 pg/mL] and tacrolimus group [130.7 pg/mL] (* $p<0.05$),

tacrolimus [130.7 pg/mL] and costimulatory blockade [16.83 pg/mL] group (* $p<0.05$).

IFN-γ: there is significant difference in serum IFN-γ levels on:

<u>Day 7</u>, between PiF [79.61 pg/mL] and:

no immunosuppression group [9.8 pg/mL], (*$p<0.05$)

tacrolimus group [6.67 pg/mL], (*$p<0.05$)

costimulatory blockade group [2.82 pg/mL], (*$p<0.05$),

<u>Day 14</u>, between PiF [134.3 pg/mL] and:

no immunosuppression group [10.09 pg/mL], (***$p<0.001$),

tacrolimus group [12.28 pg/mL], (***$p<0.001$),

costimulatory blockade group [15.84 pg/mL], (***$p<0.001$),

<u>Day 28</u>, between PiF [857 pg/mL] and:

no immunosuppression group [15.44 pg/mL], (***$p<0.001$)

tacrolimus group [12.64 pg/mL], (***$p<0.001$)

costimulatory blockade group [13.18 pg/mL], (***$p<0.001$)

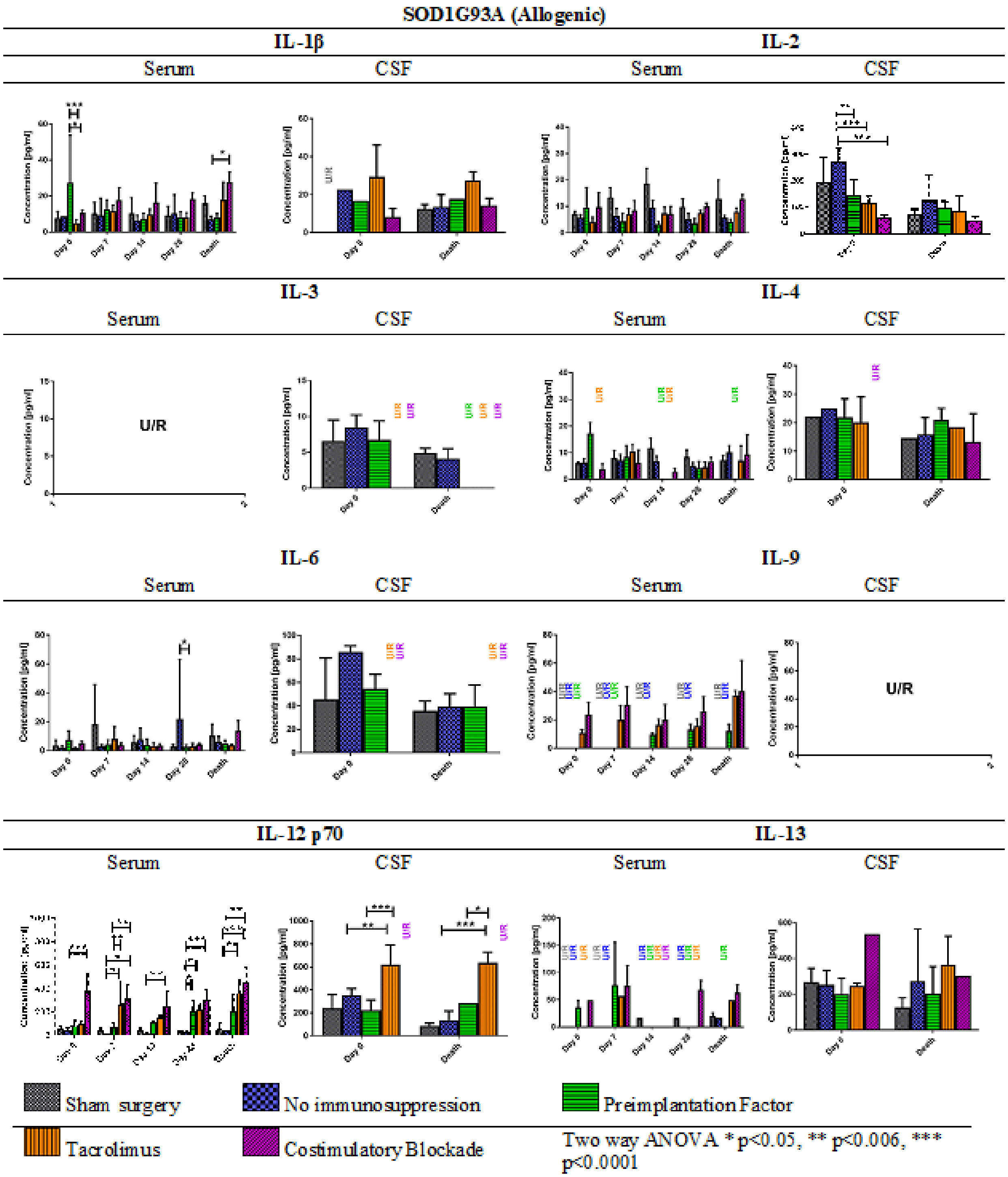

SOD1G93A (Allogenic)
IL-1β
IL-2
Serum
CSF
IL-3
IL-4
U/R
IL-6
IL-9
IL-12 p70
IL-13
Concentration [pg/ml]
Day 0
Day 7
Day 14
Day 28
Death
Sham surgery
No immunosuppression
Preimplantation Factor
Tacrolimus
Costimulatory Blockade
Two way ANOVA * p<0.05, ** p<0.006, *** p<0.0001

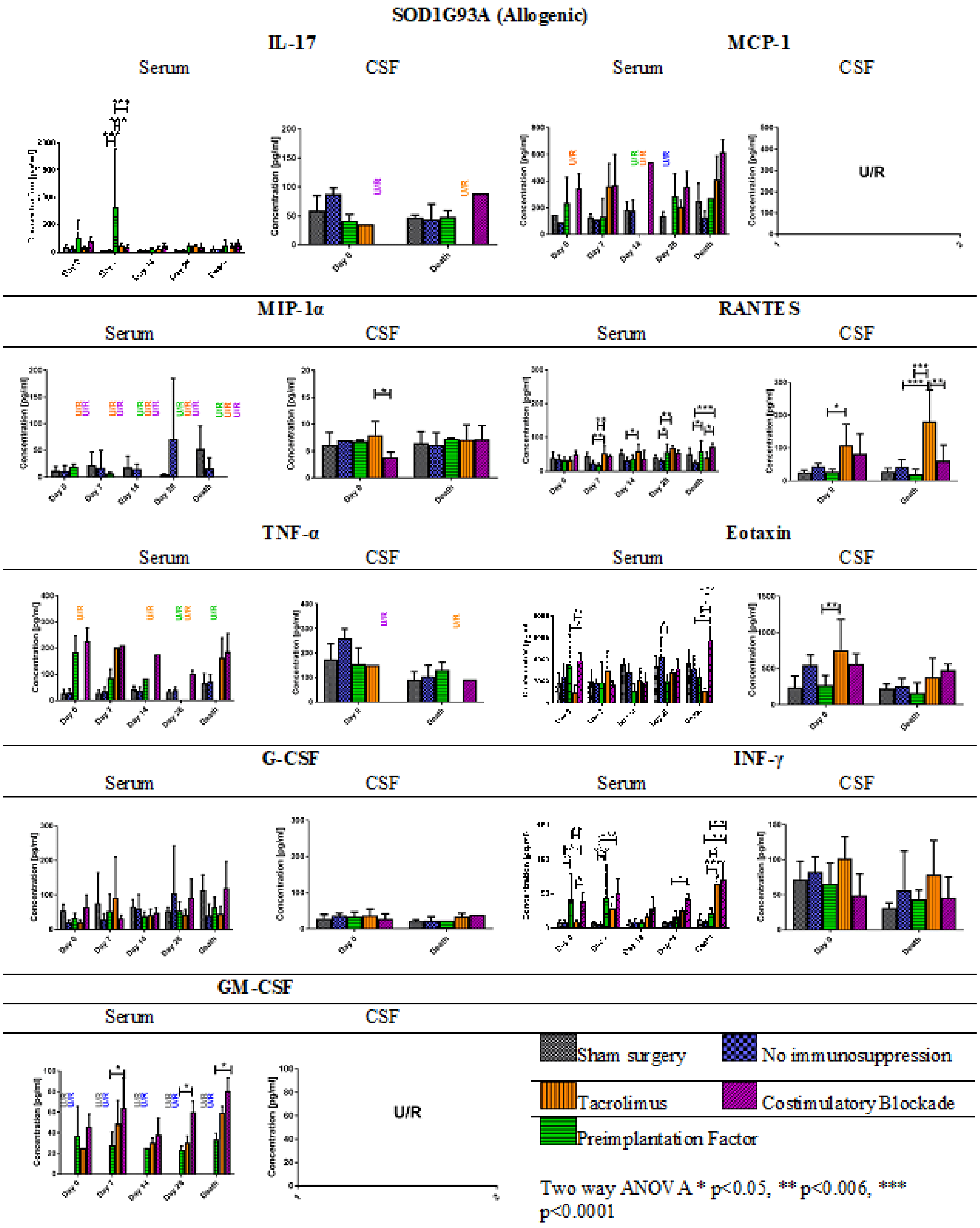

Fig. S3. The picture presents multiplex ELISA in serum and CSF of SOD1 G93A mice in various immunomodulatory and immunosuppressive regimens. Two-way ANOVA; error bars: SEM; n = 2-6 samples per experimental group. Sham group was excluded from statistic calculations.

IL-1β: there is significant difference in serum IL-1β levels on the day of death between:

no immunosuppression [7.05 pg/mL] and costimulatory blockade groups [27.58 pg/mL] (*p <0.05),

preimplantation factor [27.36 pg/mL] and tacrolimus [4.77 pg/mL] (*** p< 0.001),

preimplantation factor [27.36 pg/mL] and costimulatory blockade groups [10.59 pg/mL] (* p<0.05).

IL-2: there is no significant difference in IL-2 levels between groups in serum. In CSF, the significant difference was visible on day 0 between no immunosuppression [271.9 pg/mL] and:

PiF [146 pg/mL] group (** p<0.01),

Tacrolimus [116.1 pg/mL] group (*** p<0.001),

Costimulatory blockade group [62.88 pg/mL] (*** p<0.001),

IL-3: the levels of IL-3 in serum was undetectable. There is no significant difference between groups in IL-3 (CSF).

IL-4: there is no significant difference in IL-4 levels between groups (serum and CSF),

IL-6: there is significant difference in IL-6 levels between no immunosuppression [22.2 pg/mL] and PiF [2.47 pg/mL] groups (* p<0.05) on day 28.

IL-9: there is no significant difference in IL-9 levels between groups (serum). In CSF the measurements were under detectable range,

IL-12 (p70): there is significant difference in serum IL-12 (p70) levels between:

day 0: no immunosuppression [39.41 pg/mL] and costimulatory blockade [379 pg/mL] groups on (*** p<0.001);

day 7: no immunosuppression [22.35 pg/mL] and tacrolimus groups [269.2 pg/mL] (* p<0.05),

no immunosuppression [22.35 pg/mL] and costimulatory blockade groups [309.7 pg/mL]

(* p<0.05),

PiF [66.28 pg/mL] and tacrolimus groups [269.2 pg/mL] (** p<0.01),

PiF [66.28 pg/mL] and costimulatory blockade groups [309.7 pg/mL] (** p<0.01),

day 14: no immunosuppression [19.42 pg/mL] and costimulatory blockade [246.2 pg/mL] groups

(** p<0.01),

day 28: no immunosuppression [26.83 pg/mL] and PiF [204.4 pg/mL] groups (* p<0.05),

no immunosuppression [26.83 pg/mL] and tacrolimus groups [224.4 pg/mL] (* p<0.05),

no immunosuppression [26.83 pg/mL] and costimulatory blockade groups [305.5 pg/mL] (*** p<0.01),

day of death: no immunosuppression [42.45 pg/mL] and tacrolimus groups [363.5 pg/mL] (** p<0.01),

no immunosuppression [42.45 pg/mL] and costimulatory blockade groups [449.1 pg/mL] (*** p<0.01),

PiF [203.8 pg/mL] and costimulatory blockade groups [449.1 pg/mL] (** p<0.01),

Levels of IL-12 (p70) in CSF on <u>day 0</u> was significantly different between:

no immunosuppression [353.3 pg/mL] and tacrolimus [619.6 pg/mL] groups (** $p<0.01$),

PiF [223.5 pg/mL] and tacrolimus [619.6 pg/mL] groups (*** $p<0.001$).

On <u>day of death</u> there was significant difference between:

no immunosuppression [135 pg/mL] and tacrolimus [636.2 pg/mL] groups (*** $p<0.001$),

PiF [281.3 pg/mL] and tacrolimus [636.2 pg/mL] groups (*$p<0.05$).

IL-13: there is no significant difference in IL-13 levels between groups (serum and CSF),

IL-17: there is significant difference in serum IL-17 levels on day 7 between PiF [830.8 pg/mL] and:

No immunosuppression group [26.39 pg/mL] (*** $p<0.001$),

Tacrolimus group [106.1 pg/mL] (***$p<0.001$),

Costimulatory blockade group [93.7 pg/mL] (*** $p<0.001$),

MCP-1: there is no significant difference in MCP-1 levels between groups (serum). In CSF the measurements were under detectable range,

MIP-1α: there is no significant difference in MIP-1α levels between groups (serum). In CSF statistically significant difference was measured between tacrolimus [7.93 pg/mL] and costimulatory blockade group [3.81 pg/mL] on day 0.

RANTES: there is a significant difference in RANTES levels in serum:

<u>Day 7</u>: no immunosuppression group [24.72 pg/mL] and tacrolimus group [53.15 pg/mL] (** $p<0.01$)

PiF [19.26 pg/mL] group and tacrolimus [53.13 pg/mL] group (** $p<0.01$).

<u>Day 14</u>: no immunosuppression [32.09 pg/mL] and tacrolimus [59.44 pg/mL] group (* $p<0.05$),

<u>Day 28</u>: no immunosuppression [31.32 pg/mL] and PiF [57.15 pg/mL] groups (* $p<0.05$),

no immunosuppression [31.32 pg/mL] and tacrolimus [69.1 pg/mL] groups (** $p<0.01$),

<u>Day of death</u>: no immunosuppression [25.33 pg/mL] and PiF [58.96 pg/mL] (*$p<0.05$),

No immunosuppression [25.33 pg/mL] and costimulatory blockade [71.92 pg/mL] (*** $p<0.001$),

Tacrolimus [40.11 pg/mL] and costimulatory blockade [71.92 pg/mL] groups (* $p<0.05$).

There was significant difference between groups measured in CSF:

<u>Day 0</u>: PiF [27.15 pg/mL] and tacrolimus [108.3 pg/mL] groups (*$p<0.05$),

<u>Day of death:</u> no immunosuppression [42.01 pg/mL] and tacrolimus [180.8pg/mL] groups (*** $p<0.001$),

PiF [19.76 pg/mL] and tacrolimus [180.8 pg/mL] groups (*** $p<0.001$),

Tacrolimus [180.8 pg/mL] and costimulatory blockade [61.12 pg/mL] (** $p<0.01$),

TNF-α: there is no significant difference in TNF-α levels between groups (serum and CSF),

Eotaxin: there is a significant difference in Eotaxin serum levels between:

<u>Day 0</u>: PiF [3511 pg/mL] and tacrolimus [989.7 pg/mL] groups (** $p<0.01$),

Tacrolimus [989.7 pg/mL] and costimulatory blockade [3915 pg/mL] groups (** $p<0.01$)

Day 28: no immunosuppression [4288 pg/mL] and PiF [2070 pg/mL] groups (*$p<0.05$),

Day of death: no immunosuppression [3205 pg/mL] and costimulatory blockade [5801 pg/mL] (*$p<0.05$),

PiF [2442 pg/mL] and costimulatory blockade groups [5801 pg/mL] (*$p<0.05$),

Tacrolimus [1141 pg/mL] and costimulatory blockade groups [5801 pg/mL] (***$p<0.001$)

In CSF the significant difference of Eotaxin levels was observed on day 0 between PiF [269.4 pg/mL] and tacrolimus [754.9 pg/mL] groups (**$p<0.01$).

G-CSF: there is no significant difference in G-CSF levels between groups (serum and CSF),

IFN-γ: there is significant difference in serum IFN-γ levels between groups:

Day 0**:** no immunosuppression [7.9 pg/mL] and PiF [40.7 pg/mL] (**$p<0.01$),

No immunosuppression [7.9 pg/mL] and costimulatory blockade [39.03 pg/mL] (*$p<0.05$),

PiF [40.7 pg/mL] and tacrolimus [9.03 pg/mL] (**$p<0.01$),

Tacrolimus [9.03 pg/mL] and costimulatory blockade [39.03 pg/mL] (*$p<0.05$),

Day 7**:** no immunosuppression [5.81 pg/mL] and PiF [44.07 pg/mL] (***$p<0.001$),

no immunosuppression[5.81 pg/mL] and costimulatory blockade [50.26 pg/mL] (***$p<0.001$),

Day 28: no immunosuppression [6.72 pg/mL] and costimulatory blockade [42.33 pg/mL] (*$p<0.05$),

Day of death**:** no immunosuppression [10.01 pg/mL] and tacrolimus [63.67 pg/mL] (***$p<0.001$),

no immunosuppression [10.01 pg/mL] and costimulatory blockade [70.44 pg/mL] (***$p<0.001$),

PiF [21.48 pg/mL] and tacrolimus [63.67 pg/mL] (*$p<0.05$),

PiF [21.48 pg/mL] and costimulatory blockade [70.44 pg/mL] (**$p<0.01$),

GM-CSF: there is no significant difference in of GM-CSF in serum between PiF and costimulatory blockade groups on:

Day 7: PiF [27.97 pg/mL] and costimulatory blockade [63.86 pg/mL] (*$p<0.05$),

Day 28: PiF [23.66 pg/mL] costimulatory blockade [60.1 pg/mL] (*$p<0.05$),

Day of death: PiF [33.59 pg/mL] costimulatory blockade [80.93 pg/mL] (*$p<0.05$),

In CSF the measurements were under detectable range,
